# Chromosome-scale *Amaranthus tricolor* genome provides insights into the evolution of the genus *Amaranthus* and the mechanism of betalain biosynthesis

**DOI:** 10.1093/dnares/dsac050

**Published:** 2022-12-06

**Authors:** Hengchao Wang, Dong Xu, Sen Wang, Anqi Wang, Lihong Lei, Fan Jiang, Boyuan Yang, Lihua Yuan, Rong Chen, Yan Zhang, Wei Fan

**Affiliations:** Guangdong Laboratory for Lingnan Modern Agriculture (Shenzhen Branch), Genome Analysis Laboratory of the Ministry of Agriculture and Rural Affairs, Agricultural Genomics Institute at Shenzhen, Chinese Academy of Agricultural Sciences, Shenzhen, Guangdong 518120, China; Guangdong Laboratory for Lingnan Modern Agriculture (Shenzhen Branch), Genome Analysis Laboratory of the Ministry of Agriculture and Rural Affairs, Agricultural Genomics Institute at Shenzhen, Chinese Academy of Agricultural Sciences, Shenzhen, Guangdong 518120, China; Guangdong Laboratory for Lingnan Modern Agriculture (Shenzhen Branch), Genome Analysis Laboratory of the Ministry of Agriculture and Rural Affairs, Agricultural Genomics Institute at Shenzhen, Chinese Academy of Agricultural Sciences, Shenzhen, Guangdong 518120, China; Guangdong Laboratory for Lingnan Modern Agriculture (Shenzhen Branch), Genome Analysis Laboratory of the Ministry of Agriculture and Rural Affairs, Agricultural Genomics Institute at Shenzhen, Chinese Academy of Agricultural Sciences, Shenzhen, Guangdong 518120, China; Guangdong Laboratory for Lingnan Modern Agriculture (Shenzhen Branch), Genome Analysis Laboratory of the Ministry of Agriculture and Rural Affairs, Agricultural Genomics Institute at Shenzhen, Chinese Academy of Agricultural Sciences, Shenzhen, Guangdong 518120, China; Guangdong Laboratory for Lingnan Modern Agriculture (Shenzhen Branch), Genome Analysis Laboratory of the Ministry of Agriculture and Rural Affairs, Agricultural Genomics Institute at Shenzhen, Chinese Academy of Agricultural Sciences, Shenzhen, Guangdong 518120, China; Guangdong Laboratory for Lingnan Modern Agriculture (Shenzhen Branch), Genome Analysis Laboratory of the Ministry of Agriculture and Rural Affairs, Agricultural Genomics Institute at Shenzhen, Chinese Academy of Agricultural Sciences, Shenzhen, Guangdong 518120, China; Guangdong Laboratory for Lingnan Modern Agriculture (Shenzhen Branch), Genome Analysis Laboratory of the Ministry of Agriculture and Rural Affairs, Agricultural Genomics Institute at Shenzhen, Chinese Academy of Agricultural Sciences, Shenzhen, Guangdong 518120, China; Guangdong Laboratory for Lingnan Modern Agriculture (Shenzhen Branch), Genome Analysis Laboratory of the Ministry of Agriculture and Rural Affairs, Agricultural Genomics Institute at Shenzhen, Chinese Academy of Agricultural Sciences, Shenzhen, Guangdong 518120, China; Guangdong Laboratory for Lingnan Modern Agriculture (Shenzhen Branch), Genome Analysis Laboratory of the Ministry of Agriculture and Rural Affairs, Agricultural Genomics Institute at Shenzhen, Chinese Academy of Agricultural Sciences, Shenzhen, Guangdong 518120, China; Guangdong Laboratory for Lingnan Modern Agriculture (Shenzhen Branch), Genome Analysis Laboratory of the Ministry of Agriculture and Rural Affairs, Agricultural Genomics Institute at Shenzhen, Chinese Academy of Agricultural Sciences, Shenzhen, Guangdong 518120, China

**Keywords:** *Amaranthus tricolor*, Joseph’s-coat, Chinese spinach, *Amaranthus*, whole-genome duplication

## Abstract

*Amaranthus tricolor* is a vegetable and ornamental amaranth, with high lysine, dietary fibre and squalene content. The red cultivar of *A. tricolor* possesses a high concentration of betalains, which has been used as natural food colorants. Here, we constructed the genome of *A. tricolor*, the first reference genome for the subgenus *Albersia*, combining PacBio HiFi, Nanopore ultra-long and Hi–C data. The contig N50 size was 906 kb, and 99.58% of contig sequence was anchored to the 17 chromosomes, totalling 520 Mb. We annotated 27,813 protein-coding genes with an average 1.3 kb coding sequence and 5.3 exons. We inferred that *A. tricolor* underwent a whole-genome duplication (WGD) and that the WGD shared by amaranths occurred in the last common ancestor of subfamily Amaranthoideae. Moreover, we comprehensively identified candidate genes in betalain biosynthesis pathway. Among them, *DODAα1* and *CYP76ADα1*, located in one topologically associated domain (TAD) of an active (A) compartment on chromosome 16, were more highly expressed in red leaves than in green leaves, and *DODAα1* might be the rate-limiting enzyme gene in betalains biosynthesis. This study presents new genome resources and enriches our understanding of amaranth evolution, betalains production, facilitating molecular breeding improvements and the understanding of C4 plants evolution.

## Introduction

Major grain crops, such as rice, corn and wheat, and staple vegetable crops, such as potato, tomato and lettuce, supply calories and nutrition to humankind. However, with the development of our society, trend towards dietary homogenization worldwide^1^ and its negative consequences for the health of humans,^2^ such as diabetes, hypertension and obesity, imply that we urgently need other more nutritious vegetables rich in essential minerals, vitamins and other micronutrients important for healthful diets. As underutilized species, amaranths with unique and unparallel nutritive value, referring to species from the *Amaranthus* genus, largely planted in Asia and the Americas.^3^ Amaranths’ resilience to different climate conditions and C4 photosynthesis enable them to be cultivated in environmentally sustainable way and to have a higher photosynthetic rate. With dietary fibre, mineral content (such as iron, magnesium and calcium) and essential amino acids, vegetable amaranths help to reduce bad cholesterol, improve eyesight and prevent anaemia.^4^ However, grain amaranths attracted much of attentions for previous researches, and vegetable group received relatively insufficient study.

*Amaranthus tricolor*, also known as Joseph’s coat and Chinese spinach, is a C4 eudicot in the genus *Amaranthus*, family Amaranthaceae, order Caryophyllales. Plants in the genus *Amaranthus* can be classified as grain, vegetable, ornamental and weedy amaranths.^4^*A. tricolor* is both a nutritive vegetable and graceful ornamental amaranth. It has a well-balanced essential amino acid composition with high lysine content, dietary fibre and squalene with biological and pharmacological activities (such as reducing the risk of cancer through antitumor activity and lowering cholesterol levels in humans).^5–7^ This plant is also a rich source of minerals such as calcium, iron and zinc.^8^ The leaf pigments in *A. tricolor* show radical scavenging activity and high antioxidant potentials.^9^ Moreover, *A. tricolor* is a medicinal plant with many antimicrobial peptides.^10,11^ It is a major leafy vegetable in South and Southeast Asia, and also cultivated in East, West and Southern Africa.^3^ Chinese cuisine traditionally uses *A. tricolor* (xiàncài) as a standalone steamed or boiled vegetable dish. Due to most sought-after nutrients, *A. tricolor* is a suitable candidate for expanding our narrow vegetable base.

Betalains are tyrosine-derived pigments that correspond to universally phenylalanine-derived anthocyanins, in most Caryophyllales plants.^12^ There are two types of betalains: red-violet betacyanins and yellow betaxanthins. Betalains contribute to many essential functions in plants, such as attracting pollinators and dispersers and resisting drought and salinity stress.^13,14^ In industry, betalains are used as natural food colorants.^15,16^ Artificial synthesized food colour additives, such as the most abundant food colorants in the world Red 40 and Yellow 6, are environmental risk factors for carcinogenic or mutagenic effects^17^ and can induce colitis in the conditions of immune dysregulation.^18^ Thus, we need natural colorants to replace synthesized dyes imperatively. Moreover, intermediates of betalain biosynthesis have important medical use; for example, *L*-DOPA is used for treating Parkinson’s disease. *A. tricolor* cultivars have low or high betalain content, providing an opportunity to study the molecular bases of betalain production.^19^ In *A. tricolor*, a few genes for betalain biosynthesis have been cloned^20^ and studied by transcriptome sequencing.^21,22^ Given the time and space specificity of gene expression, transcriptome can hardly capture comprehensive functional genes of betalain biosynthesis.

Amaranthaceae comprises two subfamilies, Chenopodioideae and Amaranthoideae, with approximately 2,050–2,500 species in 180 genera.^23^ In Chenopodioideae, genomes of some common species, such as highly nutritious grain (*Chenopodium quinoa*), sugar production crop (*Beta vulgaris*) and leafy vegetable (*Spinacia oleracea*), have been sequenced, expanding our understanding of Chenopodioideae evolution and contributing to breeding at the molecular level.^24–26^ In Amaranthoideae, genome-scale studies have focused on plants of the genus *Amaranthus* and this genus, commonly known as pigweed, includes three subgenera: *Acnida*, *Albersia* and *Amaranthus*.^27^ Plants in *Acnida* are dioecious, and plants in the other two subgenera are monoecious. Recently, plants in both the subgenus *Amaranthus* and *Acnida* have been subjected to genome studies, including species *Amaranthus cruentus*,^28^*Amaranthus hypochondriacus*,^29^*Amaranthus tuberculatus*,^30^*A. hybridus* and *Amaranthus palmeri*.^31^ Thus, the lack of a genome for plants in *Albersia* has restricted studies on the evolution and biology of these plants. In this work, we presented a genome assembly for *A. tricolor*, the first reference genome for the subgenus *Albersia* in the genus *Amaranthus*. We also provided high-quality gene annotation and performed phylogenomic and transcriptomic analyses to explore the evolution of the genus *Amaranthus* and the mechanism of betalain biosynthesis in *A. tricolor*.

## Materials and Methods

### Plant materials, DNA and RNA sequencing

The *A. tricolor* cv. Red and cv. Green were grown on the farm of Agricultural Genomics Institute at Shenzhen, Chinese Academy of Agricultural Sciences, Shenzhen, China. For PacBio HiFi sequencing (Pacific Biosciences, California, USA), the Qiagen DNeasy Plant Mini Kit (Qiagen, Hilden, Germany) was used to extract high-quality genomic DNA from 30 days old tender fresh leaves of one plant for cv. Red and cv. Green, separately. The quality of DNA was checked by 0.75% agarose gel electrophoresis, Nanodrop and Qubit fluorimeter (Thermo Fisher, Massachusetts, USA). SMRTbellTM Express Template Prep Kit 2.0 (Pacific Biosciences, California, USA) was used to construct a 15-kilobase DNA SMRTbell library and PacBio Sequel II platform (Pacific Biosciences, California, USA) was used for sequencing. Pbccs v6.0 (https://github.com/PacificBiosciences/pbbioconda, October 2022, date last accessed) was used to generate circular consensus sequence from subreads. For Illumina genomic sequencing, a library of 500 bp insert length was prepared by following standard protocols of Illumina. Paired-end reads (paired-end 150, PE150) were sequenced on Illumina NovaSeq 6000 (Illumina, San Diego, USA). For Hi–C sequencing of cv. Red, the fresh leaves of cv. Red were shredded and cross-linked with 2% formaldehyde followed by digesting DNA with MboI enzyme and biotin-labelling the ends of fragments. Then, fragmented DNA was ligated and sheared, and the biotin-labelled fragments were enriched with streptavidin beads, used to build sequencing library and sequenced on Illumina HiSeq 2500 (PE150 bp) (Illumina, San Diego, USA). For genomic high-molecular-weight (HMW) DNA extraction associated with Oxford Nanopore (ONT) sequencing, fresh leaf of 4 weeks-old seedlings of cv. Red was collected and HMW gDNA was extracted from these leaves with Qiagen DNeasy Plant Mini Kit. An ultra-long ONT library was prepared with Ligation Sequencing Kit (SQK-LSK109, Nanopore, Oxford, UK) and was sequenced on MinION platform (Nanopore, Oxford, UK). Guppy v3.2.10 (Nanopore, Oxford, UK) was used to call bases. Moreover, for full-length transcriptome (PacBio Iso-Seq) sequencing of cv. Red, high-quality RNA was extracted by Qiagen kit from root, stem, leaf and flower tissues of both mature plant and young seedlings, and the standard protocol was used to construct a sequencing library with insert size 0.5–6 kb. High-quality cDNA was sequenced on PacBio Sequel II platform. For Illumina RNA sequencing, we collected four tissues from both cultivars. Among them, three tissues are from red part, green part and the boundary of both red and green part from leaves of cv. Red, and 1 tissue is from leaves of cv. Green, respectively. Each sample has four replicates and was sequenced on Illumina NovaSeq 6000.

### Genome assembly

To estimate genome size, we used Kmerfreq (https://github.com/fanagislab/kmerfreq) to count K-mer frequency based on Illumina genomic reads and the idea of GCE^32^ to get an estimated genome size. For primary contig assembly, we used hifiasm v0.14.2^33^ with parameters ‘-z 20 -D 20 -r 15 -t 24 -l0’ to assemble circular consensus sequences (CCS) high-quality reads. To filter organelle genomic and contaminated contigs, we downloaded 403,174 prokaryote and 23,229 organelle genomes from NCBI and used minimap2 v2.20 with identity > 0.95 and coverage > 0.95. In addition, we used Benchmarking Universal Single-Copy Orthologs (BUSCO) v5.2.2^34^ with the lineage dataset Embryophyta from Orthologs Database (OrthoDB) v10 to assess gene completeness.

To improve continuity, we used Nanopore ultra-long reads to scaffold contigs. We used minimap2 v2.21 to map Nanopore ultra-long reads to contigs with ‘-ax map-ont’ and filtered reads that mapped on only one contig to get reads connecting two different contigs. After that, we filtered alignments that mapped on the inner of two contigs and assigned a ‘head’ or ‘tail’ tag to each connected contig. Based on this result, we summed reads counts for the same contig pairs. We used scaffold_by_trueCtgContact.pl from EndHiC^35^ with parameters ‘--contacts 2 –reciprocalmax’ to get a GFA (Graphical Fragment Assembly) file that recorded contigs relationship. The GFA assembly graph was simplified (with simplify_gfa.pl) by removing those branch edges and breaking circular edges at the position with the weakest linkage. Then, we used cluster_and_classify_GFA.pl and order_and_orient_GFA.pl from EndHiC to cluster, order and orient contigs in GFA graph. To get confident scaffolds, we filtered out scaffolds that are shorter than 2 Mb and kept them as contigs. For each contig pair on a scaffold, we used the median of distance from the alignment of multiple reads connecting them as estimated gap size. Here, we got scaffold assembly based on Nanopore ultra-long reads alignment.

For Hi–C chromosome level scaffolding, we used ALLHiC_pip.sh (v0.9.8) in the ALLHIC package,^36^ with exception of omitting contig correction step. After that, we identified telomeres on the chromosome scaffolds based on Tandem repeats finder (TRF) results and telomere sequence of ‘CCCTAAA’ and ‘TTTAGGG’, and manually curated some contigs position in chromosome scaffolds by Juicebox,^37^ based on Hi–C heatmap and telomeres’ information.

### Repeats analysis

Tandem repeats finder (TRF) v4.09^38^ was used to identify tandem repeats in assembly with parameters ‘2 7 7 80 10 50 2000 -d -l 10 -h’. Based on the result of TRF, we detected possible centromeres on the chromosomes, using repeat unit size 126 and repeat length longer than 0.7 Mb as cut-off. The centromere regions of plants were composed of centromeric tandem repeats (satellite DNA) and transposable elements in the flanking region.^39^ However, the repeat unit sequences are very complex and vary a lot among different lineages,^40^ which is out of the scope of this study and needs further investigations. We also used the result of TRF to compare the differences of tandem repeats and AT tandem repeats (tandem repeats with ‘AT’ or ‘TA’ as repeat unit) for closely related plants. For transposable elements (TE) identification, both structure intact and fragmented TEs were annotated by Extensive *de-novo* TE Annotator (EDTA) v1.9.9^41^ and RepeatModeler v2.0.1.^42^ First, we used EDTA to produces a non-redundant TE library for annotation of structurally intact and fragmented elements. Then we used RepeatMasker v4.1.2 with parameters ‘-nolow -no_is -norna -engine ncbi -gff -lib’ to mask TE families based on the TE library created by EDTA. Then, we used RepeatMasker to identify known TEs with plant TE library in RepBase database v26.05. Later, we used RepeatProteinMask v4.1.2 to mask protein TEs in genome. We used RepeatModeler with default parameters to identify non-LTR retrotransposons and any unclassified TEs that are missed by structure-based TE identification method. We used transposable elements representation learner (TERL^43^) to classify the unclassified TE families in the TE library generated by RepeatModeler. Then we used RepeatMasker to mask the classified TEs in library from RepeatModeler and TERL. We merged all TE annotation, removed redundancy and get the final TE annotation. In addition, we also performed TE annotation for *A. tuberculatus*, *A. hybridus* and *A. palmeri*.

### Gene prediction

To predict genes, we used AUGUSTUS with hints on gene structures, such as intron, exon, part of exon, part of CDS and gene start, derived from Iso-seq, RNA-seq and protein homology. For PacBio Iso-seq full-length transcriptome sequencing of cv. Red, we used ccs v6.0.0 with parameters ‘--min-rq 0.9’ to generate CCS high quality reads, used lima with parameters ‘--isoseq --peek-guess’ to clip primer sequence. And then, isoseq3 v3.4.0 was used to refine and cluster CCS reads to get high quality transcripts. Based on the high-quality full-length transcripts, we used GMAP v2021-03-08 to map them onto genome assembly and the result file was filtered and transferred into hints file by blat2hints.pl in AUGUSTUS.^44^ For homology searches, we used exonerate v2.4.0 with parameters ‘--model protein2genome --percent 50’ to map proteins of *Amaranthus hypochondriacus*, *Arabidopsis thaliana*, *Chenopodium quinoa*, *Spinacia oleracea*, *Beta vulgaris* and Swiss-Port reviewed proteins of Embryophyta onto the genome. Alignments with frameshift were filtered. Each query should have three targets at most and low score alignments were filtered by in-house exonerate_filterFS_bestN.pl with parameters ‘--bestn 3 --bestr 0.5’. After filtering, align2hints.pl in BRAKER v2.1.2 was used to generate hints file. For Illumina RNA-seq analysis, STAR v2.7.10 was used to map reads onto the genome. The result bam file was filtered by filterBam in AUGUSTUS with parameters ‘--uniq --paired –pairwiseAlignments’. After sorting by samtools, bam2hints in AUGUSTUS was used to generate hints file with parameters ‘—intronsonly’. To avoid over masking the genome, we did not mask repeats shorter than 200 bp. After comparison of the training parameters derived from the full-length transcriptome (Iso-Seq) and BUSCO by setting coyote tobacco as reference species, we chose configure files generated by BUSCO v5.2.2 as the species-specific gene prediction models for *A. tricolor*, because we can get a more complete gene set using the BUSCO derived gene prediction models. BUSCO was run with embryophyta_odb10 as lineage dataset, with AUGUSTUS as gene predictor and tobacco as AUGUSTUS species on the genome model. Based on these gene prediction parameters, integrated hints from RNA-seq, Iso-seq and homology search and repeats soft-masked genome, AUGUSTUS v3.4.0 was used with parameters ‘--hintsfile=hintsfile.gff --gff3=on --alternatives-from-evidence=true --softmasking=on --codingseq=on’ to predict alternative transcripts of genes.

### Functional annotation

To assign gene function for the gene set, we used InterProScan v5.52-86^45^ to annotate gene functions and get Gene Ontology (GO) terms. We also aligned the protein sequences of genes with KEGG, NR and Swiss-Prot databases by diamond, an alternative replacement of blast, using 1e−5 as a cutoff and got the best hit. We used PlantTFDB v5.0^46^ (http://planttfdb.gao-lab.org/prediction.php) to annotated transcription factors and PRGdb v3.0^47^ to annotate plant disease resistance genes. Moreover, tRNAscan-SE v2.0^48^ was used to find transfer RNAs (tRNAs) with default parameters and cmscan from infernal v1.1.4^49^ was used to find other non-coding RNAs (ncRNAs) based on Rfam v14.^50^ RNAmmer v1.2^51^ with parameters ‘-S euk -m lsu,ssu,tsu’ was used to annotate 8S, 18S and 28S ribosomal RNA (rRNA). For methylation annotation, we employed nanopolish^52^ to call methylation according to Nanopore data. Firstly, minimap2 v2.24^53^ was used to map reads onto the genome with parameters ‘-x map-ont’. Nanopolish call-methylation was used to detect methylated bases at CpG sites with parameters ‘--methylation cpg’ based on reads, bam and genome files. Calculate_methylation_frequency.py from nanopolish was used to calculate methylation frequency. In addition, we also performed tRNA and rRNA annotation for *A. tuberculatus*, *A. palmeri*, *A. hypochondriacus*, *A. hybridus*, *S. oleracea* and *C. quinoa*.

### Evolution analysis

For orthogroup construction, we collected genomes of 15 species (Table S28), including *Aldrovanda vesiculosa*,^54^*Amaranthus cruentus*,^28^*A. hypochondriacus*,^29^*A. hybridus*,^31^*A. tuberculatus*,^30^*Amaranthus palmeri*,^31^*Amaranthus tricolor*, *Arabidopsis thaliana* (TAIR10.1), *Beta vulgaris*,^25^*Chenopodium quinoa*,^24^*Dianthus caryophyllus*,^55^*Fagopyrum tataricum*,^56^*Hylocereus undatus*,^57^*Simmondsia chinensis*^58^ and *Spinacia oleracea*.^26^ We used OrthoFinder v2.5.2^59^ with parameters ‘-M msa -A mafft -T fasttree -1 -y -S diamond_ultra_sens’ to build orthogroups. Fifty percent of all genes were in orthogroups with 24 or more genes (G50 was 24) and were contained in the largest 4,700 orthogroups (O50 was 4,700). OrthoFinder called Species Tree from All Genes (STAG^60^) to build an unrooted species tree and used Species Tree Root Inference from Gene Duplication Events (STRIDE^61^) to root this tree. To verify the species tree, RAxML-NG^62^ was employed to construct a species tree with parameters ‘--model LG+G8+F --tree pars 10 --bs-trees 100 --outgroup Arabidopsis_thaliana’ using *Arabidopsis thaliana* as outgroup.

To estimate divergence times among branches of the species tree, we used MEGA-CC^63^ using calibration time 75–100 Mya between *C. quinoa* and *F. tataricum* from TimeTree (http://www.timetree.org), 8 gamma distribution and LG with Freqs. (+F) model according to the multiple sequence alignment and the species tree. Subsequently, Computational Analysis of gene Family Evolution (CAFE) v5^64^ was used to identify gene family expansion and contraction with parameter ‘-k 3’ according to the time tree.

### Identification of WGD

To detect syntenic gene blocks, we employed MCScanX^65^ taking diamond blast ultra-sensitive results from the previous OrthoFinder running as input to find collinear genes. We used duplicate_gene_classifier and dot_plotter in MCScanX to classify duplicated genes and plot dot plots. Circos^66^ was used to plot circos plot. Based on collinear genes, KaKs_Calculator v2.0^67^ with parameters ‘-m GMYN’ was used to estimate synonymous substitution rate (*Ks*) for orthologue genes.

### Genome mining for betalain synthesis genes

To find genes for betalain biosynthesis, we collected public genes from *B. vulgaris*, *Cleretum bellidiforme*, *A. tricolor*, *Mirabilis jalapa* and *C. quinoa* (ADH: AST16041.1, AST12931.1, AST12935.1, AST16042.1, AST12926.1, AST12930.1; B5GT: CAB56231.1, AJY59053.1, AAL57240.1, UGY86975.1, AJY59055.1; cDOPA5GT: BAD91804.1, BAD91803.1, QOP57917.1, AJY59054.1; CYP76AD: AUZ41887.1, QOP57914.1, AZC85900.1, XP_10691493.1, XP_10695803.2, XP_10692295.1, I3PFJ5, P0DKI2; DODA: Q70FG7, I3PFJ3, I3PFJ9, QOP57916.1, QOP57915.1; MYB AET43456.1, AET43457.1, ANU06195.1, AVI04855.1; UDPGT: NP_1345948.1, XP_21754077.1, XP_3607533.2) and used BLASTP^68^ to search homology counterparts of these public genes on proteins of the gene set using evalue 1e−5 as cut-off. The resulting alignment was manually checked and exonerate^69^ was used to map these public genes on the genome to detect missing genes in the gene set, resulting in no new candidate genes in the genome. Then, we used MUSCLE v3.8.31^70^ to do multiple sequence alignment (MSA) with candidate genes of *A. tricolor* and reference genes, and checked them manually. pyBoxshade (https://github.com/mdbaron42/pyBoxshade) was used to show MSAs. We also checked annotated domains from InterProScan results. The homology genes of other plants in Caryophyllales were found by analysing orthogroups from OrthoFinder results. Based on MSAs, fasttree v2.1.11^71^ with parameters ‘-lg -gamma’ was used to create gene trees for different kinds of genes.

### RNA-seq data analysis

For RNA-seq analysis, STAR^72^ was used to map Illumina reads onto the genome of *A. tricolor*. Reads count of each gene was acquired from the mapping results and was used to obtain differentially expressed genes by DESeq2^73^ in R v.4.2.0. Furthermore, we used our in-house PERL program to calculate Transcripts Per Million (TPM). For heatmaps of gene expression, we used pheatmap and RColorBrewer packages of R to draw figures based on TPM.

### TAD calling and A/B compartment analysis

To study the three-dimensional genome of *A. tricolor*, we used HiC-Pro^74^ to map Hi–C data onto chromosome-scale genome with bin sizes 20,000 and 500,000 bp. For TAD identification, using 20 kb resolution matrix, hicConvertFormat from HiCExplorer^75^ package with parameters ‘--correctForMultipleTesting fdr’ was used to convert matrix format into h5 format; hicCorrectMatrix diagnostic_plot was used to identify bins with low and high read coverage; hicCorrectMatrix correct with parameters ‘--filterThreshold -1.5 3.6’ was used to balance raw matrix; hicFindTADs with parameters ‘--thresholdComparisons 0.005 --correctForMultipleTesting fdr’ was used to call TADs, resulting in 1,437 TADs. pyGenomeTracks^76^ with parameters ‘--region Chr16:4,500,000-7,500,000’ was used to draw figures near key genes of betalain biosynthesis. For 3D genome structures, chromosomes can be partitioned to distinct A and B compartments, which are enriched for active and repressed chromatin, respectively. A/B compartment annotation is largely in accordance with the euchromatin/heterochromatin landscapes of the genome.^77^ For A/B compartment analysis, hicCorrectMatrix correct with parameters ‘--filterThreshold -1.6 1.4’ was used to balance the raw matrix of 500 kb resolution; hicPCA was used to compute A/ B compartments; hicPlotMatrix was used to plot pearson correlation matrix. Because A compartments are active regions of chromosomes, and usually have less methylation, more gene number, higher gene expression and less Hi–C interaction, we assigned A/ B compartments according to gene number, expression level of genes, Hi–C reads count and methylation level.^77^

## Results

### Chromosome-scale genome and high-quality gene set of *A. tricolor*

**Figure 1.**
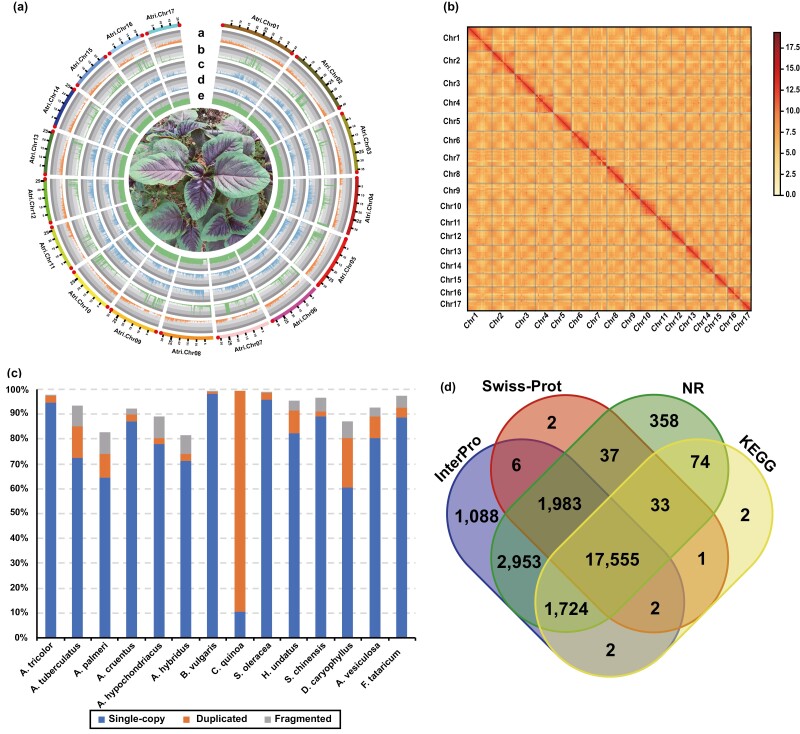
Overview of the chromosome-scale genome and high-quality gene set of *A. tricolor*. (a) Circos plot of *A. tricolor*. Pink dots on chromosome ends represent telomeres. Most chromosomes have telomeres on both ends, and only four chromosomes (Chr04, Chr06, Chr07 and Chr08) have telomeres on one end. a, gene density distribution; b, tandem repeat density distribution; c, transposable element (TE) *Gypsy* density distribution; d, TE *Copia* density distribution; e, AT percentage distribution. The window size is 100 kb, and a picture of *A. tricolor* is in the centre of this plot. (b). Genome-wide contact matrices between each pair of the 17 chromosomes for *A. tricolor* from Hi–C data. The resolution (bin size) is 500 kb, and the colour value represents base 2 logarithm of the link number (log2[link number]), where the link number is the number of Hi–C links falling into the two analysed genomic bins. (c). Benchmarking Universal Single-Copy Orthologs (BUSCO) assessment of gene sets for 14 species in Caryophyllales. Single-copy means complete and single-copy BUSCOs; duplicated means complete and duplicated BUSCOs; fragmented means fragmented BUSCOs. The lineage dataset used here is Embryophyta from OrthoDB v10. The gene set of *A. tricolor* showed the highest complete BUSCO ratio among amaranths. (d) Venn plot of functional annotation based on different databases for the gene set of *A. tricolor*. We used a web tool to draw this figure (http://bioinformatics.psb.ugent.be/webtools/Venn) (See online version for colour figure).

To acquire a high-quality reference genome for *A. tricolor*, we integrated PacBio HiFi, Nanopore ultra-long and Hi–C data to construct a chromosome-level assembly (Supplementary Table S1). Firstly, 23.3 Gb (37 X) Illumina reads were used to analyze K-mer frequency, and the analysis showed that the plant exhibited a low heterozygosity rate, and the genome size was estimated to be 562.5–687.5 Mb (Supplementary Fig. S1a). The genome size estimated from PacBio HiFi reads was 418.5–511.5 Mb (Supplementary Fig. S1b), which is much smaller than the genome size estimated from Illumina reads, implying that HiFi data may lose some sequence compared to Illumina data. Then, hifiasm^33^ assembled 28.1 Gb (45×) HiFi reads with an N50 length of 14 kb into primary contigs with 2,544 contigs, assembly size 519 Mb and an N50 size of 906 kb. The assembly size was smaller than the genome size estimated by Illumina data, showing that some genomic regions were lost in the assembly and could not be resolved by the current algorithm. To improve the continuity of the assembly, we used 52.0 Gb (83X) Nanopore ultra-long reads with an N50 length of 48 kb to scaffold the primary contigs into scaffolds with 2,412 scaffolds, assembly size 520 Mb and an N50 size of 2 Mb (Supplementary Table S2). Based on 78.6 Gb (126 X) Hi–C reads, ALLHIC^36^ anchored 99.58% of the scaffold sequences to the 17 chromosomes (Fig. 1a and b). The final chromosome-scale genome consisted of a 520 Mb assembly with an N50 size of 32 Mb, with the longest being 45 Mb (Table 1 and Supplementary Table S2, Fig. S2). The genome assembly contained 30 of 34 (88.24%) telomeres on the ends of chromosomes, with only four chromosomes with telomeres on one end, which is much more than that of closely related plants in Amaranthaceae (Fig. 1a, Table 1, Supplementary Fig. S3 and S4). In contrast to *Arabidopsis thaliana*, which has a 178 bp centromere repeat unit, and *Oryza sativa*, which has a 155 bp centromere repeat unit,^78^*A. tricolor* has a 126 bp repeat unit with the longest comprising 3.12 Mb (Table S5). All chromosomes contained centromeres.

**Table 1.** Genome features of *A. tricolor* and closely related plants in Amaranthaceae.

	*A. tricolor*	*A. tuberculatus*	*A. cruentus*	*A. palmeri*	*A. hypochondriacus*	*A. hybridus*	*B. vulgaris*	*S. oleracea*	*C. quinoa*
Genome assembly									
Estimated genome size by K-mer (Gb)	0.6	0.7	0.4	0.4	0.4	0.5	0.7	1.0	1.5
Total assembly size (bp)	520,084,113	688,987,999	370,913,848	411,927,395	395,806,076	411,833,878	566,550,431	869,946,296	1,333,551,035
Number of contigs	2,544	2,514	1,608	628	787	640	71,208	215,349	4,212
Contig N50 size (Mb)	0.91	1.74	1.02	2.54	1.15	2.26	0.03	0.02	1.79
Number of scaffolds	48	16	625	16	16	48	40,246	78,263	3,487
Scaffold N50 size (bp)	31,692,477	43,088,275	21,701,286	26,298,281	24,364,990	24,954,950	34,941,034	319,471	3,844,283
% of sequences anchored to chromosomes	99.58%	99.8%	98.5%	99.89%	98%	99.8%	84.70%	47.00%	85%
% of telomeres been assembled	88.24%	12.5%	17.65%	0.00%	12.5%	25.00%	0.00%	0.00%	0.00%
Number of chromosomes	17	16	17	16	16	16	9	6	18
GC content	31.93%	34.94%	33.08%	33.18%	32.71%	33.01%	36.14%	37.82%	36.80%
BUSCO complete rate of the genome	97.5%	97.5%	94.1%	96.8%	96.4%	98.5%	98.0%	97.6%	98.3%
Genome annotation									
Length and % of tandem repeats (Mb)	119 (23%)	37 (6%)	14 (4%)	27 (7%)	20 (5%)	22 (5%)	27 (5%)	59 (7%)	145 (11%)
Length and % of TE sequences (Mb)	354 (68%)	477 (69%)	215 (58%)	240 (58%)	194 (48%)	245 (59%)	252 (42%)	618 (74%)	854 (64%)
Number of tRNA genes	1,289	1,174	926	1,132	1,031	1,153	1,297	2,384	2,877
Number of rRNA genes	4,113	776	325	227	99	2,026	232	345	1,316
Number of protein-coding gene models	27,813	44,992	25,248	26,506	23,677	23,820	24,351	25,609	49,138
Total CDS size and % in genome (Mb)	35 (7%)	39 (6%)	29 (8%)	31 (7%)	25 (6%)	28 (7%)	32 (6%)	34 (4%)	63 (5%)
BUSCO complete rate of gene set	97.5%	85.32%	89.84%	74.16%	80.3%	74.0%	99.07%	98.70%	99.44%
Mean gene length	4,893	3,247	4,323	3,151	4,872	4,472	5,552	5,046	4,752
Mean CDS length	1,246	863	1,140	1,154	1,068	1,174	1,315	1,308	1,280
Mean CDS number	5.30	3.15	4.85	5.07	4.87	4.79	4.92	4.90	4.91

*Note*: We performed transposable element (TE) annotation for *A. tuberculatus*, *A. hybridus* and *A. palmeri*, and we also performed tRNA and rRNA annotation for *A. tuberculatus*, *A. palmeri*, *A. hypochondriacus*, *A. hybridus*, *S. oleracea* and *C. quinoa*, because their genome paper did not analyze them.

For assembly assessment, Benchmarking Universal Single-Copy Orthologs (BUSCO) were used. According to OrthoDB v10 of the lineage dataset Embryophyta (*n* = 1,614), the genome contained 97.5% (1,574) complete BUSCOs, and 2.9% (47) complete and duplicated BUSCOs, which were comparable to the BUSCOs of other genomes in Caryophyllales (Supplementary Table S6 and Supplementary Figure S3). This result indicated that our genome assembly showed satisfactory quality.

For gene prediction, we integrated evidence from full-length transcriptome (Iso-seq), short reads transcriptome (RNA-seq) and homologous proteins. From the Iso-seq data, 249,671 ‘exon part’ hints, 677,852 exon hints and 793,689 intron hints were obtained (Supplementary Table S7). On the basis of protein homology, 1,052,608 ‘CDS part’ hints, 835,926 intron hints and 116,933 gene start hints were acquired (Table S8). From the RNA-seq data, 435,738 intron hints were obtained (Table S9). Based on the above hints and the soft-masked genome, AUGUSTUS annotated 27,813 gene models with a mean CDS length of 1.3 kb and mean CDS number 5.30 (Figure S4 and Table S10). The total CDS length was 35 Mb, which was comparable to that of other amaranths (Table 1). The BUSCO complete rate of the gene set (97.5%) was the same as that of the genome assessment and was higher than the BUSCO scores of gene sets in other amaranths (Figure 1c), indicating that our gene set was of great quality. For functional annotation, KEGG, NR, InterPro and Swiss-Prot assigned functions to 19,393 (69.73%), 24,717 (88.87%), 25,313 (91.01%) and 19,619 (70.54%) predicted genes, respectively (Figure 1d, Table S11). In total, homology information on 25,820 (92.83%) genes was obtained from one of these databases. In addition, we annotated 354 Mb (68%) transposable elements (TEs), 1,289 tRNAs, 4,113 rRNAs and 2,075 other non-coding RNAs in *A. tricolor*, comparable to other plants (Table 1, Supplementary Tables S12 and S13). Notably, DNA and LTR type TEs are the most abundant TEs, accounting for 36.95% (192 Mb) and 28.42% (148 Mb) of the genome, respectively (Supplementary Table S14). Using PlantTFDB v5.0,^46^ we annotated 1,337 transcription factors (TFs) belonging to 57 TF families (Supplementary Table S15). Using PRGdb v3.0,^47^ we also annotated 1,240 plant disease resistance genes in *A. tricolor* (Table S16).

### AT-tandem repeats lead to shorter contigs of *A. tricolor*

**Figure 2.**
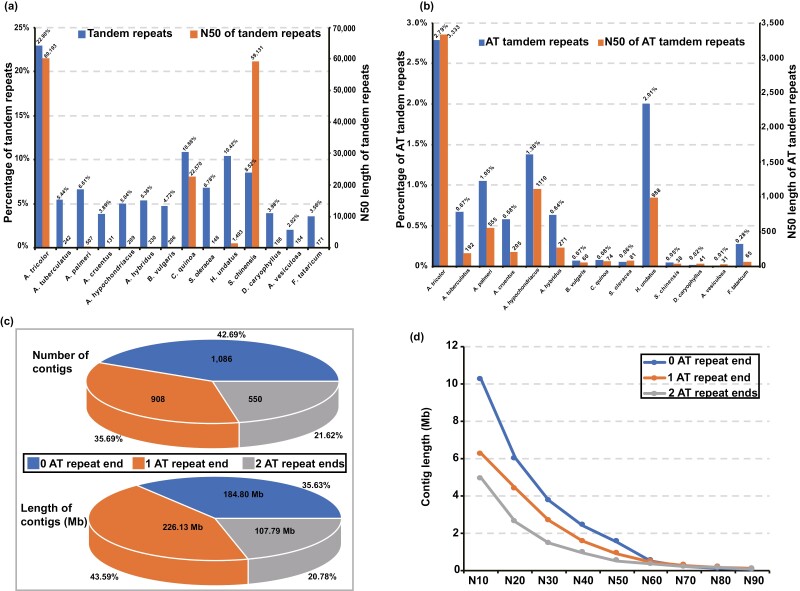
Tandem repeat features of *A. tricolor*. (a) Histogram of tandem repeat content and N50 length of tandem repeats in Caryophyllales plants. The tandem repeats in *A. tricolor* are more numerous and longer than those in others plants. (b) Histogram showing the AT tandem repeat content and N50 length of AT tandem repeats in Caryophyllales plants. The AT tandem repeats in *A. tricolor* are more numerous and much longer than those in others plants. (c) Pie plot of contig numbers and lengths for different kinds of contigs. We classified contigs into three categories based on the number of contig ends with AT tandem repeats. (d) Nx plot of contig length for three different contig categories as classified in (c). Contigs with more AT tandem repeat ends tended to be shorter than those with fewer AT tandem repeat ends. After sorting contig lengths from longest to shortest, we added up lengths and the sum making up N0% of the assembly length were used as *x*-axis.

To investigate the reason for less continuous contig assembly compared to recently published genomes built by PacBio HiFi data,^79,80^ we compared tandem repeats (several copies of exact or approximate repeat sequences concatenated head-to-tail) of Caryophyllales plants. *A. tricolor* had 119 Mb tandem repeats, accounting for 22.90% of the genome, which was much more than 37 Mb (5.44%) in *A. tuberculatus*, 14 Mb (3.89%) in *A. cruentus* and 27 Mb (6.61%) in *A. palmeri* in the genus *Amaranthus* (Figure 2a, Table S17). Moreover, the N50 length of tandem repeats was 60 kb, which was much longer than that of *A. tuberculatus* (0.24 kb), *A. cruentus* (0.13 kb) and *A. palmeri* (0.51 kb). Considering the genome of *A. tricolor* had the lowest GC percentage (only 31.93%) among closely related species (Supplementary Table S18), we assumed that this feature may affect contig assembly. Taking the longest contig as an example, most bases of the head and tail of this contig were AT tandem repeats, and the coverage of HiFi reads was very low (Supplementary Figure S5). Recently, the Telomere-to-Telomere (T2T) consortium found that PacBio HiFi reads showed AT bias, similar to the GC bias of Illumina reads.^81^ Therefore, we suggested that a high level of AT tandem repeats in *A. tricolor* led to less continuous contig assembly and smaller assembly size compared with the genome size estimated from Illumina reads. Then, we found that AT tandem repeats accounted for 2.79% of the genome assembly of *A. tricolor*, which was the highest percentage among all sequenced Caryophyllales plants (Figure 2b, Supplementary Table S19). At the same time, the N50 length of the AT tandem repeats in *A. tricolor* was the longest among Caryophyllales species.

To confirm our speculation, we analysed contigs with AT tandem repeats at the end. We classified contigs into three categories based on the number of AT tandem repeats at the end of each contig. Nine hundred eight contigs with AT tandem repeats at either end and 550 contigs with AT tandem repeats at both ends accounted for 57.31% of all the contigs, meaning that more than one-half of the contigs might have been terminated because of AT tandem repeats (Figure 2c, Supplementary Table S20). In total, contigs with AT tandem repeats at one or two contig ends summed to 333.92 Mb (64.37%) of the genome, indicating that AT tandem repeats affected the majority of assembly (Figure 2c). The contigs without AT tandem repeats ends accounting for 42.69%, making up 35.63% of the genome sequence, were shorter on average compared to the other contigs. However, the N50 length of contigs with AT tandem repeats at 0, 1 and 2 contig ends were 1.53 Mb, 0.91 Mb and 0.51 Mb, respectively, showing that contigs without AT tandem repeats at the end tended to be longer and contigs with AT tandem repeats at 1 end were longer than contigs with AT tandem repeats at 2 contig ends (Figure 2d). This trend also occurred in the PacBio HiFi reads, that is, the reads with more AT tandem repeats at the ends were shorter than those without or with less AT tandem repeats at the ends (Supplementary Table S21). Thus, these results supports the conclusion that an abundance of long AT tandem repeats of the *A. tricolor* genome and the AT bias of PacBio HiFi data together led to more fragmented contig assembly and smaller assembly size compared to the genome size estimated from Illumina data.

### The last common ancestor of amaranths is estimated to have emerged 5.73 Mya

To study the evolutionary history of the genus *Amaranthus*, we combined gene sets of 14 closely related plants from six families in Caryophyllales and considered *A. thalina* as the outgroup. The 14 plants included nine plants in Amaranthaceae: *A. tricolor*, *A. tuberculatus*, *A. palmeri*, *A. cruentus*, *A. hypochondriacus*, *A. hybridus*, *B. vulgaris*, *S. oleracea* and *C. quinoa*; one plant in Caryophyllaceae: *D. caryophyllus*; one plant in Cactaceae: *H. undatus*; one plant in Simmondsiaceae: *S. chinensis*; one plant in Droseraceae: *A. vesiculosa*; and one plant in Polygonaceae: *F. tataricum* (Supplementary Table S10). Among the plants in Amaranthaceae, the subfamily Amaranthoideae included *A. tricolor*, *A. tuberculatus*, *A. palmeri*, *A. cruentus*, *A. hypochondriacus* and *A. hybridus*, and the subfamily Chenopodioideae included *B. vulgaris*, *S. oleracea* and *C. quinoa*. All the plants in the subfamily Amaranthoideae studied here belonged to the genus *Amaranthus*. However, *A. tricolor* belonged to subgenus *Albersia*; *A. cruentus*, *A. hypochondriacus* and *A. hybridus* belonged to subgenus *Amaranthus*; and *A. tuberculatus* and *A. palmeri* belonged to subgenus *Acnida*.^27^ Our data included nearly all Caryophyllales families with published genomes, except for pokeweed in Phytolaccaceae because of low genome quality.

**Figure 3.**
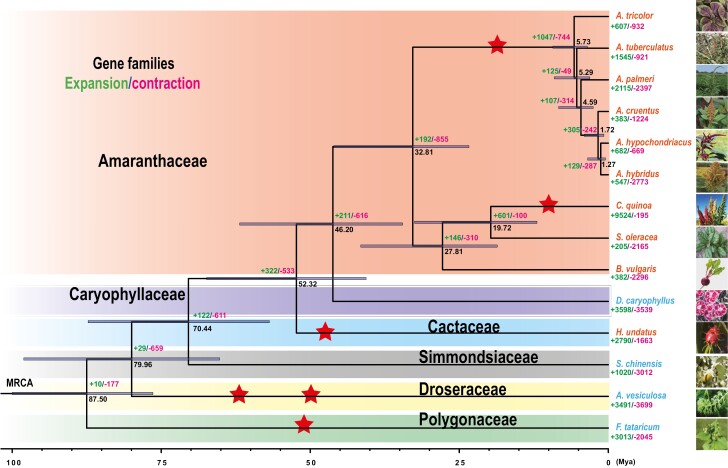
A phylogenetic tree showing topology, divergence times and expansion and contraction of gene families for 14 plants in Caryophyllales. The numbers in green and magenta at the branches indicate the expansion and contraction of gene families, respectively. The numbers in black show divergence times and red pentagrams indicate whole-genome duplications. The blue horizontal bars show 95% confidence intervals of the inner nodes. Members of the families Amaranthaceae, Caryophyllaceae, Cactaceae, Simmondsiaceae, Droseraceae and Polygonaceae are in red, blue, cyan, black, yellow and green backgrounds, respectively. The names of species in orange and cyan indicate that the species can produce betalains and anthocyanins, respectively. A picture of each plant is on the right (See online version for colour figure).

Based on the above dataset, OrthoFinder^59^ assigned 429,016 genes (94.3% of total) to 25,428 orthogroups, and there were 5,917 orthogroups with all species present. Most of the genes (89.0–98.8%) in all these plants were assigned to orthogroups (Supplementary Table S22). Using 1,004 orthogroups with a minimum of 80.0% of species carrying single-copy genes in any orthogroup, OrthoFinder built a rooted species tree (Figure 3). Then, we employed RAxML-NG^62^ to reconstruct a new species tree to verify the species tree (Supplementary Figure S6). These two strategies for estimating the species tree resulted in the same topology and very similar sequence evolution rates (branch lengths).

The relationships among plants in the subfamily Chenopodioideae were highly congruent with previous studies based on genomic analyses.^28,82^ The relationships among plants in subfamily Amaranthoideae were also highly consistent with an earlier study based on four concatenated, partitioned nuclear genes.^83^ Moreover, *A. tuberculatus* and *A. palmeri* are weedy amaranths from subgenus *Acnida*. Our results indicated that the two plants in subgenus *Acnida* did not form one clade (Figure 3), which may be associated with the faster substitution rate of *A. palmeri* (Supplementary Figure S6). In addition, the Angiosperm Phylogeny Group (APG) IV^23^ placed the families of Droseraceae and Polygonaceae into one clade, but this clade showed low bootstrap support (Supplementary Figure S7). Former plastid phylogenomic analyses had shown that Droseraceae and Polygonaceae families formed one taxon.^84^ However, our comparative genomic analyses based on orthogroups from whole-genome data showed that the Droseraceae family is not sister to the Polygonaceae family, and that Polygonaceae is a basal family and is sister to the last common ancestor of other families studied (Amaranthaceae, Caryophyllaceae, Cactaceae, Simmondsiaceae and Droseraceae) (Figure 3). The family Polygonaceae, including food crops (buckwheat and tartary buckwheat), traditional Chinese herbal medicine (golden buckwheat and *Fallopia multiflora*) and natural colorant (*Polygonum tinctorium*), was classified as an order Polygonales by the Cronquist system, but was classified as a family in the order Caryophyllales by the APG IV. Because the APG system is now widely used by botanists and since the relationships within Caryophyllales are complex, the evolutionary history of plants in this order needs more investigation and may be updated in the future.

To study divergence time, we also constructed a time tree. The results showed that *A. tricolor* diverged from the last common ancestor of other amaranths studied here 5.73 million years ago (Mya), from *B. vulgaris* 32.81 Mya, and from *D. caryophyllus* 46.20 Mya (Figure 3). Moreover, to investigate expansion and contraction of gene families of 14 plants, we used Computational Analysis of gene Family Evolution (CAFE5)^64^ with gamma rate categories to model gene gain and loss across the species tree. The results showed that 607 gene families were expanded and 932 gene families were contracted in *A. tricolor* compared to other plants (Figure 3). For *C. quinoa*, 9,524 gene families were expanded and were much more abundant than that of *S. oleracea* (205) because of allotetraploid character of *C. quinoa*.

### Whole-genome duplication event of *A. tricolor* shared in the last common ancestor of subfamily Amaranthoideae

**Figure 4.**
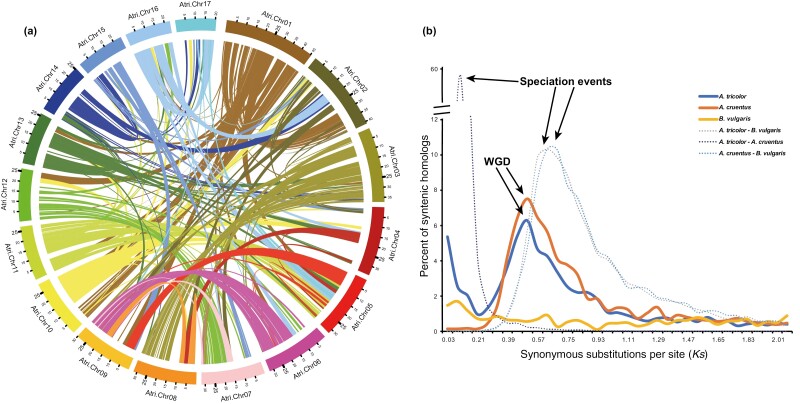
Recent whole-genome duplication of *A. tricolor*. (a). Macro-synteny plot of *A. tricolor*. The internal links are pairwise syntenic blocks, and the colours of the links are based on *A. tricolor* chromosomes. The figure shows syntenic blocks with more than seven syntenic gene pairs. (b) Homologous synonymous substitution rate per site (*Ks*) distribution for *A. tricolor*, *A. cruentus* and *B. vulgaris*. Speciation and whole-genome duplication (WGD) events are pointed out. For intra-species analysis, KaKs_calculator calculated *Ks* with the GMYN model based on the paralog gene pairs located on collinear fragments with more than five syntenic gene pairs. Inter-species analysis, in addition to collinearity, required reciprocal best ortholog gene pairs (See online version for colour figure).

To investigate the whole-genome duplication (WGD) event, we used MCScanX^65^ to detect syntenic gene blocks of the chromosome-level genome of *A. tricolor*. The classification of duplicated genes showed that 29.34% of all genes were derived from WGD/ segmental duplications, which was similar to that of the other amaranths (Supplementary Table S23). From intraspecies macro-synteny analysis, we inferred that *A. tricolor* was shaped by a recent WGD (Figure 4a). Some pairwise chromosomes such as Chr03:Chr08, Chr16:Chr17 and Chr06:Chr09, demonstrated clear macro-synteny from the WGD (Supplementary Figure S8). After the WGD, *A. tricolor* underwent violent chromosome rearrangements. In interspecies comparisons, dot plots between *A. tricolor* and *A. cruentus*, *A. palmeri*, *A. hyponchondricus* and *B. vulgaris* all supported the WGD of *A. tricolor* (Supplementary Figure S9). Chromosomes of *B. vulgaris* and chromosomes of *A. tricolor* mostly showed a 1:2 relationship, while chromosomes of *A. tricolor* and that of *A. cruentus*, *A. palmeri* and *A. hyponchondricus* mainly showed a 2:2 relationship (Supplementary Figure S9). Previous studies based on plants in subgenus *Amaranthus* inferred that a shared WGD occurred in the *Amaranthus* lineage.^28,29^ Our results indicated that this WGD occurred in all three subgenera in the genus Amaranthus. Although we detected traces of the WGD, plants in the three subgenera showed profound chromosomal structure variations that occurred after this WGD. From pairwise chromosomes comparison between *A. tricolor* and *A. cruentus*, we found that many homologous chromosomes underwent fission and fusion events (Supplementary Figure S10), and that such variations were found between *A. tricolor* and *A. hyponchondricus*, and also between *A. tricolor* and *A. palmeri* (Supplementary Figures S11 and S12).

To verify the WGD event in the *Amaranthus* lineage, we analysed the distribution of synonymous substitution rate (*Ks*) of homologous gene pairs for *A. tricolor*, *A. cruentus* and *B. vulgaris* (Figure 4b). The *Ks* peaks for divergence between *A. tricolor* and *B. vulgaris*, and between *A. cruentus* and *B. vulgaris* were all approximately 0.63, corresponding to the divergence between the subfamily Amaranthoideae and the subfamily Chenopodioideae. The *Ks* peaks for the WGD of *A. tricolor* and *A. cruentus* were all approximately 0.51. Because *A. tricolor* diverged from *B. vulgaris* approximately 32.81 Mya, we estimated that the WGD event in these amaranths occurred 26.56 Mya. Given the last common ancestor of these amaranths emerged 5.73 Mya, we supposed that the last common ancestor of the subfamily Amaranthoideae experienced this WGD.

### Structure variations between cultivar Red and cultivar Green of *A. tricolor*

To further discover the genomic difference between cv. Red and cv. Green, we assembled the genome of *A. tricolor* cv. Green and called structure variations between them. For genome assembly, we used hifiasm to assemble 25.2 Gb PacBio HiFi reads, and after filtering we obtained 3,334 contigs totalling 555 Mb with an N50 size of 570 kb (Supplementary Table S24). The slight difference in the assembly size between two cultivars may derive from the genetic difference of the two cultivars or random factors in the sequencing and assembly methods. Then, we aligned this genome with the chromosome-level genome of cv. Red and called 5,418 structural variants, totalling 5,714,483 bp (Supplementary Figure S13 and Supplementary Table S25). *A. tricolor* comprises many cultivars and genotypes,^6^ offering the opportunity for pan-genome study, which can represent the genomic sequence diversity of different cultivars and wild relatives for a species. The two genomes for both cv. Red and cv. Green of *A. tricolor* are valuable resources for future pan-genome construction and evolution studies of *A. tricolor*.

### Expression and regulation of *DODAα1* and *CYP76ADα1* affect betalain production in *A. tricolor*

**Figure 5.**
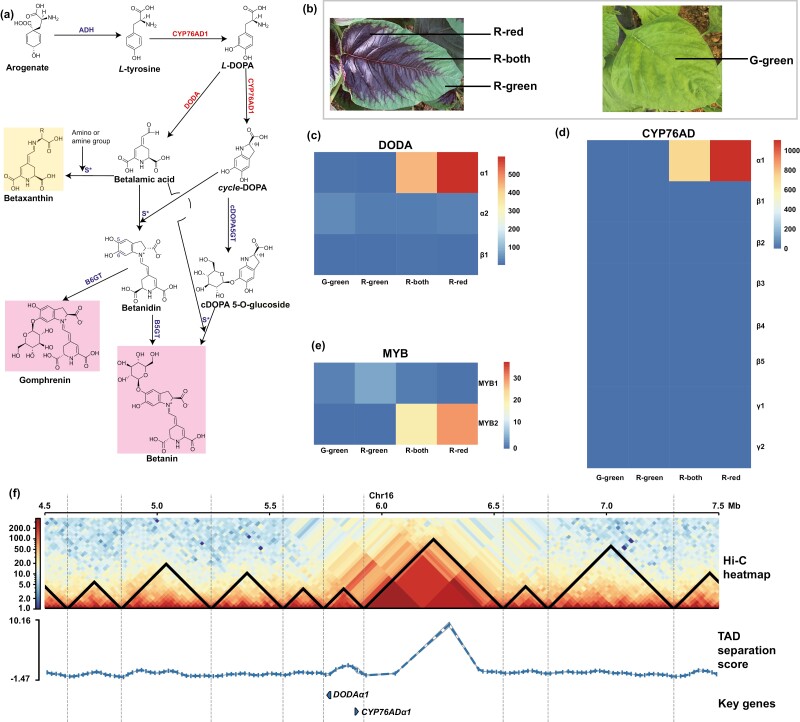
The expression and regulation of key genes in the betalain synthesis pathway influence the difference between red and green appearance of *A. tricolor*. (a) Betalain pigmentation biosynthetic pathway. The molecules with yellow and pink backgrounds are yellow betaxanthins and red–violet betacyanins, respectively. Key enzymes influencing the colour difference in *A. tricolor* are shown in red, and the other enzymes are shown in blue. S* indicates spontaneous reactions. ChemDraw v19.0 was used to generate this figure. (b) Picture of tissues used to sequence the transcriptome. R-red and R-green are sections of leaves with red and green phenotypes from cv. Red, respectively. R-both is the boundary section between R-red and R-green from cv. Red. G-green represents green leaves from cv. Green. For transcriptome sequencing, we used four biological replicates from each tissue. (c) Heatmap showing *DODA* gene expression. For each gene, the colour value represents the expression level defined by transcripts per million (TPM), and we calculated TPM by averaging the expression of four replicates. (d) Heatmap showing *CYP76AD* gene expression. For each gene, the colour value represents the expression level defined by TPM, and we calculated TPM by averaging the expression of four replicates. (e) Heatmap showing *MYB* gene expression. For each gene, the colour value represents the expression level defined by TPM, and we calculated TPM by averaging the expression of four replicates. (f) TAD appearance for a 3 Mb region on chromosome 16 at 20 kb resolution. One of the TADs in this region includes key genes of betalain biosynthesis (*DODAα1* and *CYP76ADα1*) (See online version for colour figure).

The biosynthetic pathway of betalain pigments starts with tyrosine,^85^ which comes from the shikimate pathway (Figure 5a). In *A. tricolor*, arogenate dehydrogenase (ADH) decarboxylates arogenate to generate tyrosine. Then, tyrosine is hydroxylated into *L*-DOPA by cytochrome P450 enzymes (CYP76AD), which also catalyse reactions transferring *L*-DOPA into *cycle*-DOPA. Alternatively, the enzyme *L* -DOPA 4,5-dioxygenase (DODA) cleaves the cyclic ring within *L* -DOPA in an oxidation reaction to generate intermediate 4,5-*seco*-DOPA, which is then spontaneously turned into betalamic acid. Betalamic acid can condense with an imino or amino group of an amino acid to produce yellow betaxanthins or can combine with the imino group of *cyclo*-DOPA to spontaneously give betanidin. Next, *cyclo*-DOPA 5-O-glucosyltransferase (cDOPA5GT), betanidin 5 glucosyltransferase (B5GT) and betanidin 6 glucosyltransferase (B6GT) glycosylate *cycle*-DOPA, betanidin and betanidin to give cDOPA 5-O-glucoside, betanin and gomphrenin, respectively. Then, cDOPA 5-O-glucoside spontaneously condenses with betalamic acid to generate betanin. Betanin and gomphrenin are red-violet betacyanins. These betacyanins can acquire additional glucosyl groups by UDP-glycosyltransferase (UDPGT) and other modifications, contributing to the structural diversity of betalains. In addition to these structural genes, a transcription factor R2R3-MYB controlling the expression of *DODA* and *CYP76AD1* in *B. vulgaris* was found.^86^ To identify candidate genes for betalain biosynthesis, we collected genes from closely related species, such as *B. vulgaris* and *C. quinoa*, in public databases. Using these genes as queries, we comprehensively found 3 *ADH*, 8 *CYP76AD*, 3 *DODA*, 1 *cDOPA5GT*, 6 *B5GT*, 5 *B6GT*, 3 *UDPGT* and 2 *R2R3-MYB* genes in *A. tricolor* by homology searching (Supplementary Table S26).

To investigate the factors influencing the colour difference between red and green tissues, we extracted samples from fresh leaves of *A. tricolor* cv. Red and cv. Green (G-green) for RNA-seq analyses, and each sample was sequenced in four biological replicates. The leaves of cv. Red were divided into red (R-red), green (R-green) and both (with red and green, R-both) sections (Figure 5b). For gene expression, G-green and R-green expressed at profoundly lower levels than R-both and R-red for genes *DODAα1*, *CYP76ADα1* and *MYB2* (Figure 5c, d and e). These genes were also expressed at higher levels in R-red than in R-both. Because R-red produced more betalains than other tissues^21^ and since other enzyme genes showed no obvious difference in expression, the level of gene expression for *DODAα1* and *CYP76ADα1* might have restricted the amount of betalain produced in *A. tricolor*. Additionally, *DODAα1* was the most significantly differentially expressed gene between R-green and R-both and between R-green and R-red (Data S1). Given the lower expression level of *DODAα1* compared to that of *CYP76ADα1*, we suggested that *DODAα1* may play an important role in betalain biosynthesis. Moreover, we found that *DODAα1* and *CYP76ADα1* were located near each other on chromosome 16, as reported in other Amaranthaceae species,^87^ and this region was within a topologically associated domain (TAD), as determined through three-dimensional genomic analyses with different settings (Supplementary Figure 5f and S14). The TAD containing *DODAα1* and *CYP76ADα1* was situated in an A compartment (active compartment) of this chromosome (Supplementary Figure S15 and Supplementary Table S27). Besides, three *ADH* genes regulating tyrosine synthesis were identified, and *ADHα1* was highly expressed in all four samples (Supplementary Figure S16). ADHβ enzymes are strongly feedback inhibited by tyrosine, while ADHα enzymes exhibit relaxed sensitivity to tyrosine and can produce a higher concentration of tyrosine for betalains synthesis.^88^ Thus, these results were consistent with former studies in other plants. In addition, using AlphaFold2,^89^ a state-of-the-art protein 3D structure prediction method, we predicted protein structures for these enzymes, which may help explore the molecular bases of betalain generation (Supplementary Figure S16–S23).

Based on the gene set of cv. Red, we acquired 27,414 genes for cv. Green with a mean 1.3 kb CDS length and mean number of 5.32 exons. Most of the genes involved in biosynthetic pathway of betalain pigments are the same between cv. Green and cv. Red. Furthermore, we found that a *B5GT* gene (*g8639*) of cv. Green became a pseudogene containing 28 stop codons. This gene underwent a frameshift mutation mainly caused by the insertion of an adenine residue at position 344 of the coding sequence. Another *B5GT* gene (*g27669*) of cv. Green did not mutate one base compared to that of cv. Red, however the CDS length was 1,131 bp in cv. Green, which was 180 bp shorter than that (1,311 bp) of cv. Red (Supplementary Figure S24). However, the shorter length of B5GT gene in cv. Green may be due to the incomplete assembly or annotation. These two *B5GT* genes were expressed at extremely low levels in cv. Green and might have lost their functions, which may have been caused by the low selection pressure on these genes in this cultivar. Although the other genes in the betalain synthesis pathway remained intact, the regulatory network of these genes in cv. Green may have changed compared to that of cv. Red, leading to inefficient betalain production.

## Discussion

With the development of sequencing technology, third-generation sequencing has a longer read length and higher accuracy. This will help us to better carry out whole genome sequencing, and help us to better understand biology from the genome level. *A. tricolor* is a common vegetable that is very popular in China and South-East Asia. Researches on *A. tricolor* are deficient, and genome-level study is still lacking. Here, we present a chromosome-scale genome and a high-quality gene set for *A. tricolor*. The genome assembly contains most of the telomeres and centromeres. Although the genome of *A. tricolor* contained a high percentage of long tandem repeats that could not be fully assembled by the current algorithms, especially long AT tandem repeats and the overall AT content reached 68.07%, the chromosome-level scaffold assembly was unaffected, as determined by the long-range linkage information from the Hi–C data. In the genus *Amaranthus*, plants with published genomes are grain or weedy amaranths,^28–31^ and our genome is the first genome of both vegetable and ornamental amaranth. Because of the high nutritional values and medicinal compounds of *A. tricolor*, the high-quality genome and genes will be useful in molecular-level improvements in future breeding programs. Furthermore, the transcription factors and the plant disease resistance genes we identified will be valuable resources for metabolic regulation network identification and improved breeding for disease resistance.

Previous studies reported that *A. cruentus* and *A. hypochondriacus* diverged from each other 1.45 Mya.^28^ They are two plants in the subgenus *Amaranthus*. However, to the best of our knowledge, the divergence time between subgenera of the genus *Amaranthus* had not been studied to date. We found that *Albersia* diverged from the last common ancestor of subgenera *Amaranthus* and *Acnida* 5.73 Mya, and the subgenus *Amaranthus* diverged from *Acnida* 1.72 Mya. According to the earlier study, these results are reasonable and deepen our understanding of the evolution of the genus *Amaranthus*. Moreover, a previous study reported that the ancestors of the genus *Amaranthus* shared a WGD event between 18 and 34 Mya.^29^ Based on our analyses, we estimated that this WGD occurred approximately 26.56 Mya, which is much earlier than the divergence time between the three subgenera. In line with the time tree, subfamily Amaranthoideae and subfamily Chenopodioideae diverged from each other 32.81 Mya, only 6.25 Mya before the WGD of these amaranths. These results implied that this WGD may have occurred in the last common ancestor of the subfamily Amaranthoideae. Further genomic studies of plants, other than amaranths, in the subfamily Amaranthoideae such as *Celosia argentea* and *Achyranthes bidentate* may confirm this conclusion.

Betalains are abundant in *A. tricolor*.^90^ Transcriptome and gene cloning studies revealed a few genes for the core betalain biosynthesis pathway in *A. tricolor*, but the genes for precursor tyrosine biosynthesis and downstream glucosyltransferase, such as enzymes for amaranthin generation, are still unknown.^22^ We comprehensively identified candidate genes. Among them, DODAα1 and CYP76ADα1 are enzymes critical to betalain generation, and an earlier study by qRT-PCR had found that only *CYP76ADα1* in a cv. Red was highly differentially expressed compared to cv. Green, and proposed that it played a key role in producing red betalain pigments.^22^ However, we showed that *DODAα1* was the most significantly differentially expressed gene and was expressed at lower level than *CYP76ADα1* in samples with a high concentration of betalains. Therefore, we implied that the expression of *DODAα1* may be the key factor that influences betalain contents. On the other hand, an earlier study showed that *DODAα1* and *CYP76ADα1* co-localized in the genomes of Amaranthaceae species.^28^ These two genes are also located near each other in *A. tricolor*, confirming this conclusion. Furthermore, we verified that these two genes are situated in one TAD in an A compartment of chromosome 16. Given that three-dimensional (3D) chromatin organization is highly correlated with the functionality of the genome,^77^ our results provide new insights into gene arrangement for betalain synthesis and lay the foundation for further studies on 3D genome evolution. In addition, the predicted protein structures for these enzymes will be useful for the *de novo* design of proteins that can efficiently produce betalains.

In addition to betalains, there are many other valuable compounds in *A. tricolor*. Squalene, a terpene, is an important compound in skin cosmetics and lubricant for computer disks and has shown beneficial effects on health. Compared to other plants, *A. tricolor* contains a high concentration of squalene^91^ and is thus a natural source for replacing traditional sources from marine animals, such as shark and whale. Moreover, the seeds of *A. tricolor* contain ~8% oil, which is higher than that of grain amaranths.^92^ Among the oils it produces, vitamin E is important to vision, reproduction and other aspects of human health and shows antioxidant properties mediated through two distinct kinds of molecules: tocopherol and tocotrienol. Tocotrienol is a better antioxidant than tocopherol, and the content of tocotrienol in *A. tricolor* seeds is higher than that in grain amaranths and other common vegetables such as soybean oil and peanut oil.^92^ In addition, antimicrobial peptides (AMPs), also known as host defence peptides, compose part of the innate immune system in plants and are promising compounds to treat antibiotic-resistant bacteria. Many AMPs such as Atr-AMP1,^11^ Atr-DEF2,^93^ Atr-SN1, Atr-DEF1 and Atr-LTP1^10^ have been discovered in *A. tricolor*. Therefore, our genomes and genes may help promote betalain, squalene and tocotrienol bioproduction, may rapidly predict AMPs in silico to guide approaches for treating antibiotic resistance, may contribute to studies into other agronomic and economical traits, and improve the breeding of *A. tricolor* at the molecular level to obtain better disease resistance and stress tolerance.

## Supplementary Material

dsac050_suppl_Supplementary_Material

dsac050_suppl_Supplementary_Data

## Data Availability

All the data that support this project including whole-genome sequencing data and genome resources have been deposited at GenBank with the Project ID PRJNA891371 and China National Genomics Data Center (https://ngdc.cncb.ac.cn) with the Project ID PRJCA009026. The genome assemblies, gene annotations and other resources are also available at AGIS website (ftp://ftp.agis.org.cn/~fanwei/Amaranthus_tricolor). Code for scaffolding by Nanopore ultra-long reads and for gene expression calculation from transcriptome can be found in https://github.com/whc2/A.tricolor_methods.
