# Supplementary material for: Chromosome-scale Amaranthus tricolor genome provides insights into the evolution of the genus Amaranthus and the mechanism of betalain biosynthesis
Source: DNA Res. 2022 Dec 6;30(1):dsac050. doi: 10.1093/dnares/dsac050 (PMC9847342; doi:10.1093/dnares/dsac050)
Supplement: dsac050_suppl_Supplementary_Material [file dsac050_suppl_supplementary_material.docx]

**Supplementary Information**

**Chromosome-scale *Amaranthus tricolor* genome provides insights into the evolution of the genus *Amaranthus* and the mechanism of betalain biosynthesis**

Hengchao Wang, Dong Xu, Sen Wang, Anqi Wang, et al.

**Supplementary Methods**

**Structure prediction of enzymes of betalain biosynthesis for *A. tricolor***

For protein structure prediction, AlphaFold2 (Jumper *et al.*, 2021) with parameters “--model_preset=monomer --max_template_date=2022-02-01” was employed to predict 3D structures of CYP76AD, DODA, cDOPA5GT, B5GT, B6GT, UDPGT and MYB. However, due to homodimer character of PDH, a homology protein to produce tyrosine, ADH also forms a homodimer and the structure was predicted with “--model_preset=multimer”. The prediction named “ranked_0.pdb” with highest accuracy was taken as final result. Open-source PyMOL v2.5.0 (<https://pymol.org>) was used for demonstration.

**Genome assembly and gene annotation of *A. tricolor* cv. Green**

For contig assembly of cv. Green, we used hifiasm to assembly PacBio HiFi reads and filtered organelle genomic and contaminated contigs with same settings as cv. Red, resulting in 3,334 contigs totaling 555 Mb. To validate this assembly, paired-end Illumina genomic reads was mapped onto genome of cv. Green by bwa v0.7.17 and 99.59% of reads could be mapped. We used BUSCO to assess completeness of assembly, resulting in 97.6% complete and 3.4% duplicate rate. Both results indicate our assembly cover most of genome of *A. tricolor*.

To annotate genes for cv. Green, we used Liftoff (Shumate & Salzberg, 2021) to lift over genes of cv. Red onto genome of cv. Green according to genomes of two cultivars and gene set of cv. Red, resulting in 27,414 genes in cv. Green. The BUSCO scores are 96.7% complete and 2.4% duplicate rate.

**Structure variation analysis between cv. Red and cv. Green**

To call structure variations between cv. Red and cv. Green, we used MUMmer v3.23 (Kurtz *et al.*, 2004) with “-maxmatch -l 100 -c 500” to map contig assembly of cv. Green onto reference genome of cv. Red. After that, Assemblytics (Nattestad & Schatz, 2016) (<http://assemblytics.com>) was used to find genome structure variations with default parameters. In total, we detected 5,418 variants, totaling 5.71 Mbp. Most variants with size 50-500 are insertion and deletion.

**Supplementary Figures**

**
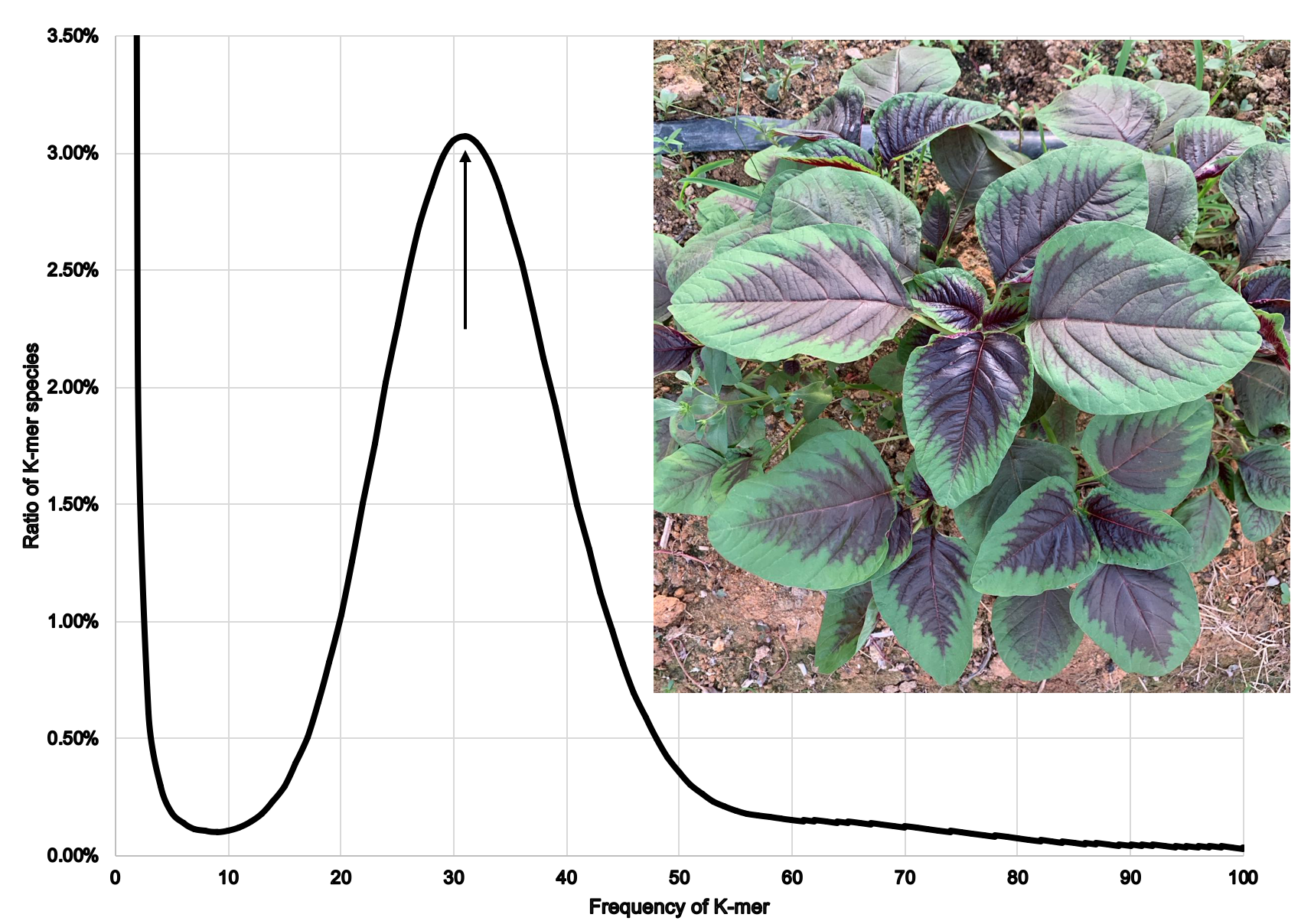
**

(b)

(a)

Figure S1. Distribution of K-mer (K = 19) frequency in sequencing reads for *A. tricolor*. The genomic sequencing data from Illumina reads (a) and PacBio HiFi reads (b) were used here. The K-mer frequency peak with arrow is the main peak, which reflects the “unique” regions in the genome. Based on the K-mer distributions, we know that this plant has low heterozygosity rate; peak values are 31 and 59, and the estimated genome sizes are 625 ± 62.5 Mb (562.5 - 687.5 Mb) and 465 ± 46.5 Mb (418.5 - 511.5 Mb) for Illumina and PacBio HiFi reads, respectively.

Figure S2. Hi-C heatmap of each chromosome for *A. tricolor*. The resolution (bin size) is 500-Kb, and color represents Log2(links number). Links number is the number of Hi-C links falling into the two analyzed genomic bins.

Figure S3. BUSCO assessment of genomes in Caryophyllales. Single-copy, Duplicated and Fragmented mean complete and single-copy BUSCOs, complete and duplicated BUSCOs, and fragmented BUSCOs, respectively. We used BUSCO v5.2.2 with lineage dataset embryophyta_odb10 to assess genomes of plants in Caryophyllales. The high duplication ratio of *A. palmeri* may be caused by heterozygous fragments in the genome. The high duplication ratio of *C. quinoa* is due to its allotetraploid genome.

Figure S4. Comparison of gene characteristics among related plant genomes. CDS stands for coding sequences. Distribution of CDS length, number of exons and intron length among the gene sets of these plants. *Atri*, *Atub*, *Apal*, *Acru*, *Ahyp*, *Ahyb*, *Cqui*, *Sole*, *Bvul*, *Dcar*, *Hund*, *Schi*, *Aves*, *Ftat*, *Atha* stands for *A. tricolor*, *A. tuberculatus*, *A. palmeri*, *A. cruentus*, *A. hypochondriacus*, *A. hybridus*, *C. quinoa*, *S. oleracea*, *B. vulgaris*, *D. caryophyllus*, *H. undatus*, *S. chinensis*, *A. vesiculosa*, *F. tataricum* and *A. thaliana*, respectively.


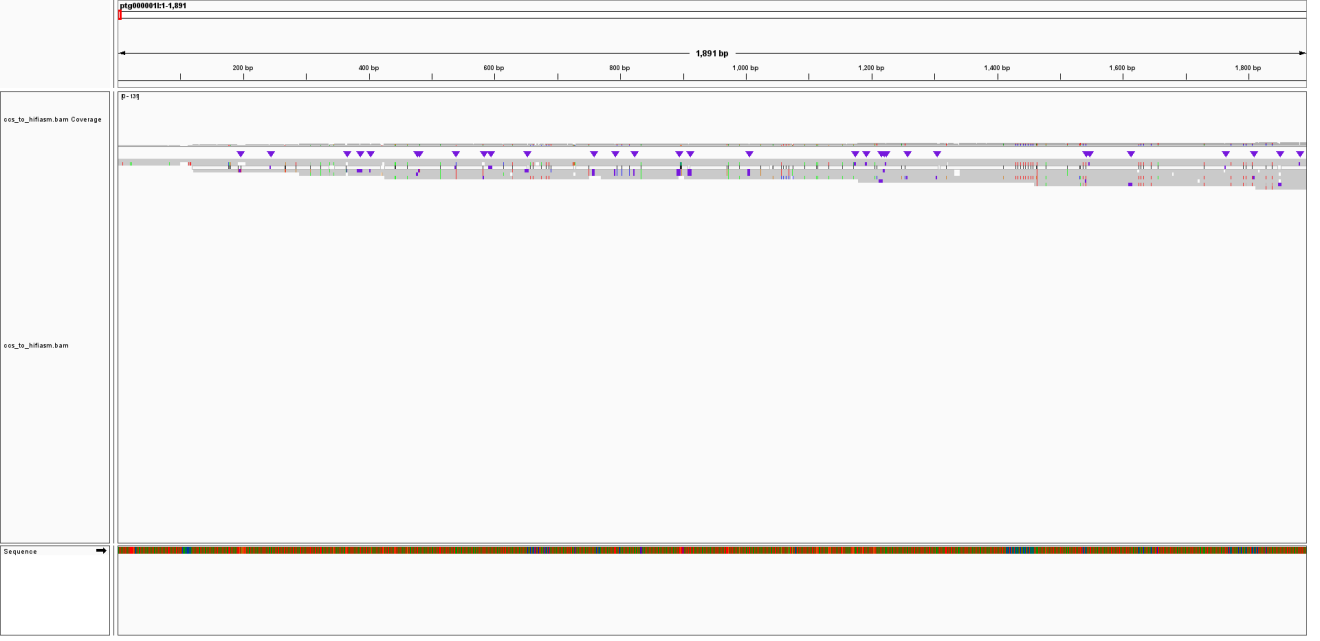


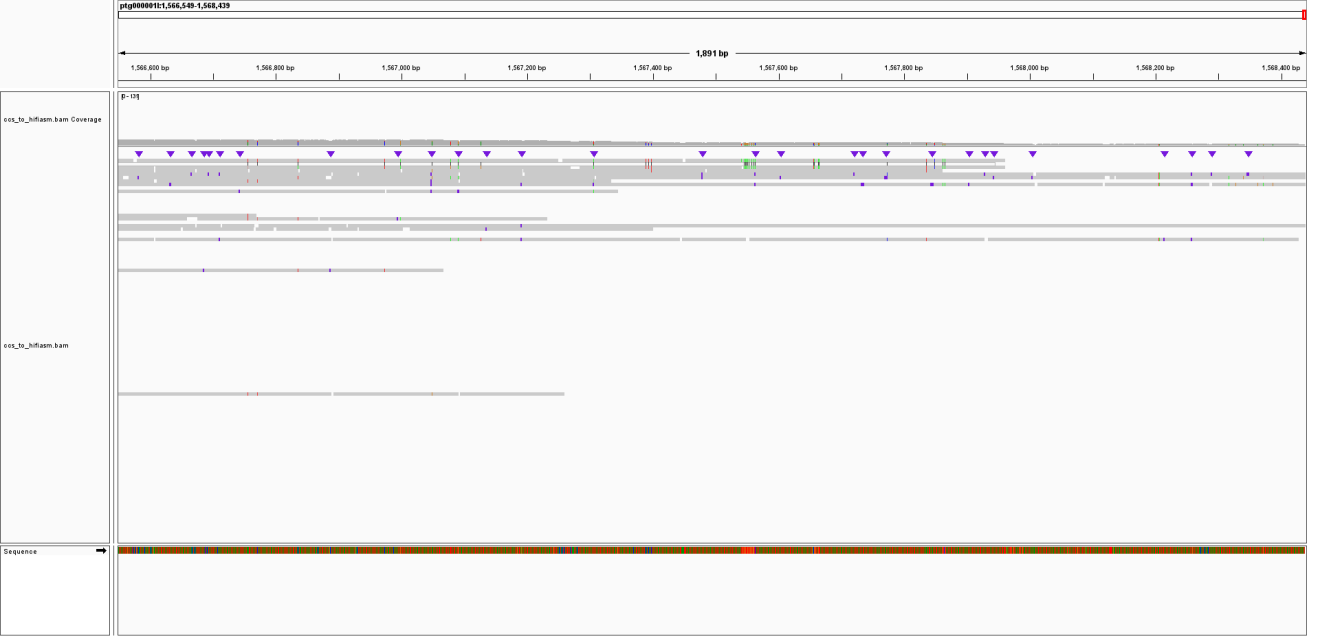


Figure S5. Screenshot of PacBio HiFi reads mapping coverage of contig ptg000001l by IGV. The red and blue color stands for base “A” and “T”, respectively. Because of AT bias, reads coverage of head and tail of contigs are lower than that of middle part of contig. The top and the bottom show the screening shoot of head and tail of this contig, respectively.

Figure S6. Species tree built by RAxML-NG. This tree have same topology and very similar branch length as tree of Figure 3. Numbers on the nodes are Felsenstein bootstrap support values.


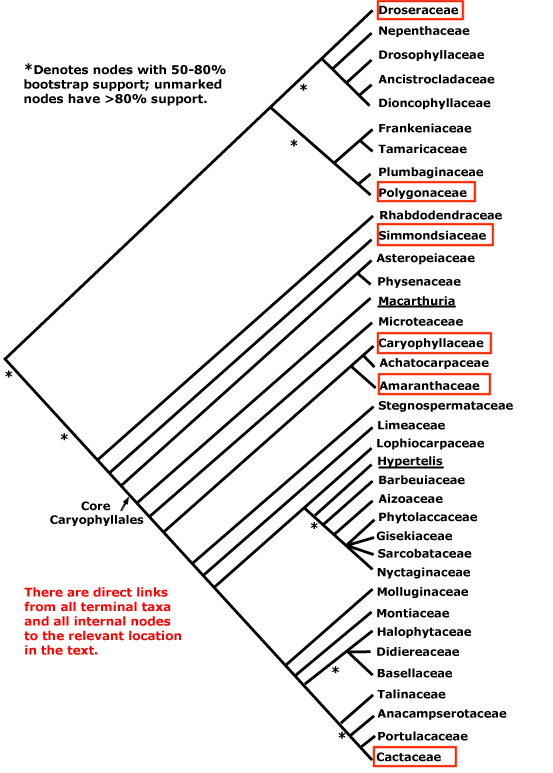


Figure S7. Evolutionary history of Caryophyllales from Angiosperm Phylogeny Group (APG) IV (<http://www.mobot.org/MOBOT/Research/APweb/welcome.html>). Data accessed on 2022.06.07 and red rectangle contains families studied in this work.


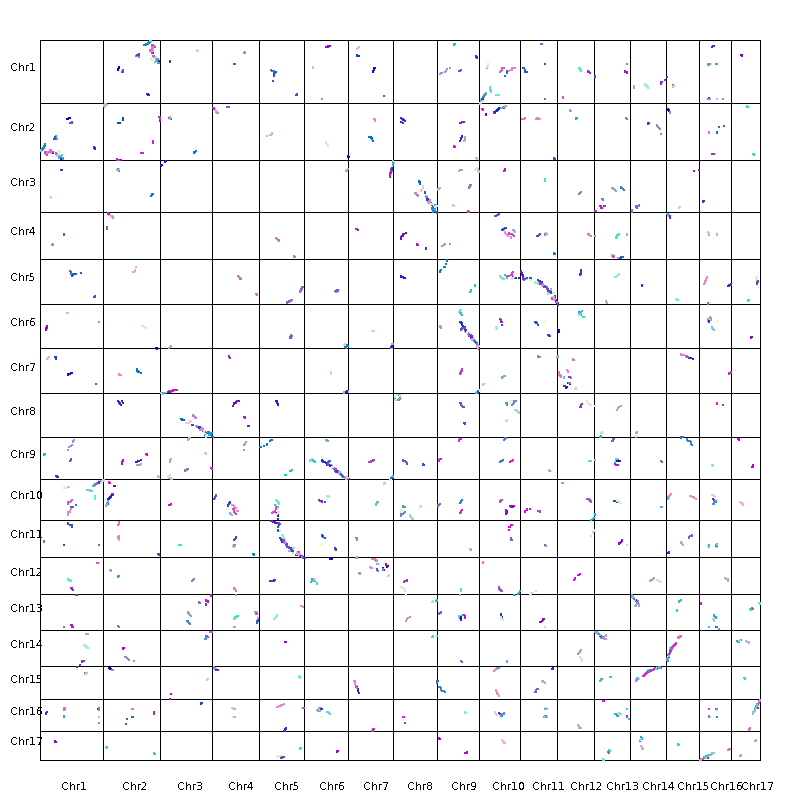


Figure S8. Dot plot of synteny blocks for *A. tricolor*. A large number of chromosomes have homology chromosomes due to whole genome duplication event.


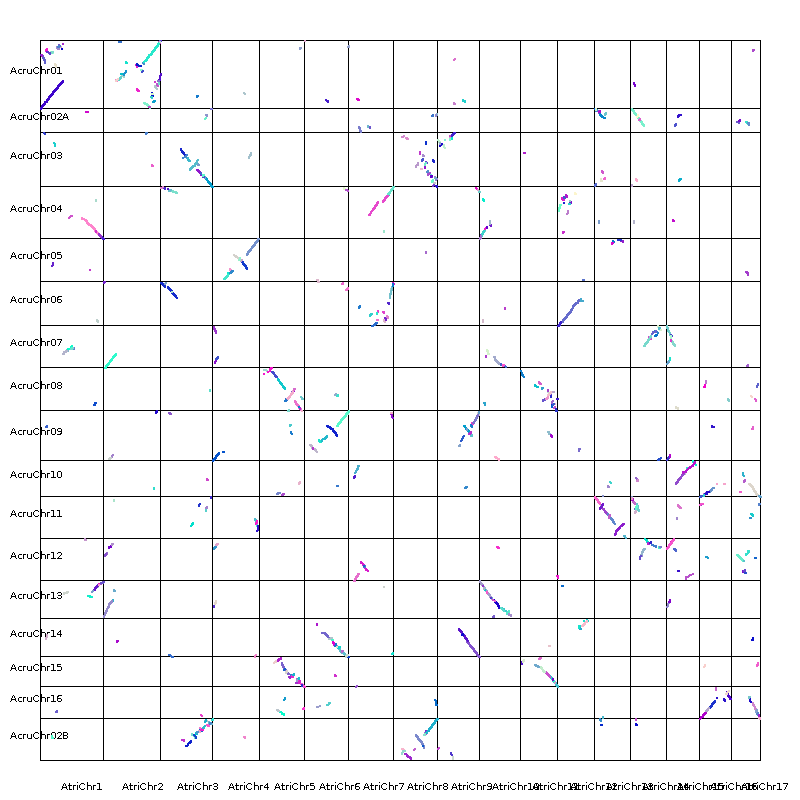

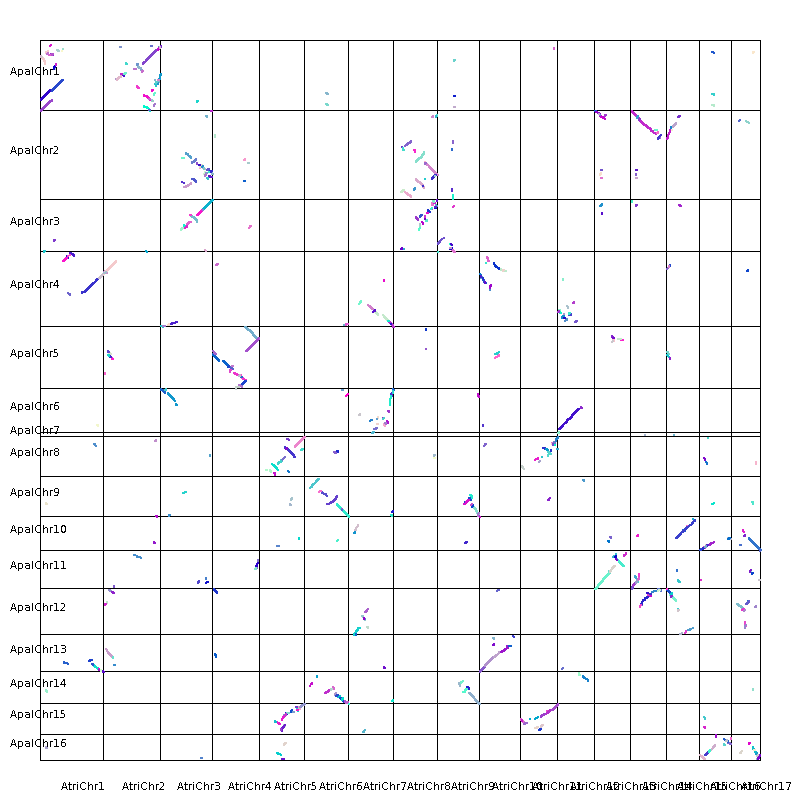


(d)

(c)

(b)

(a)


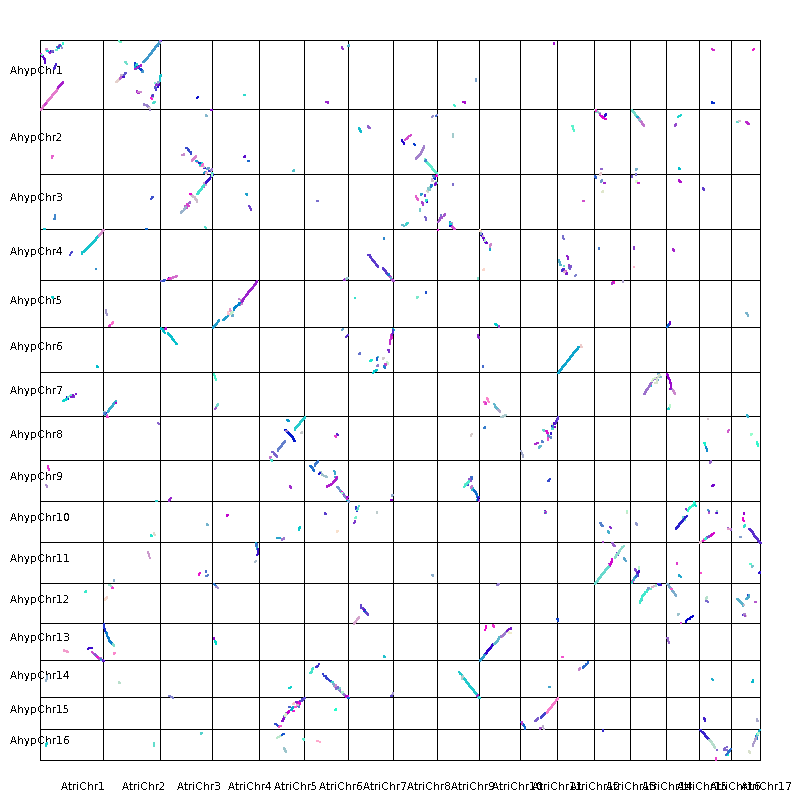

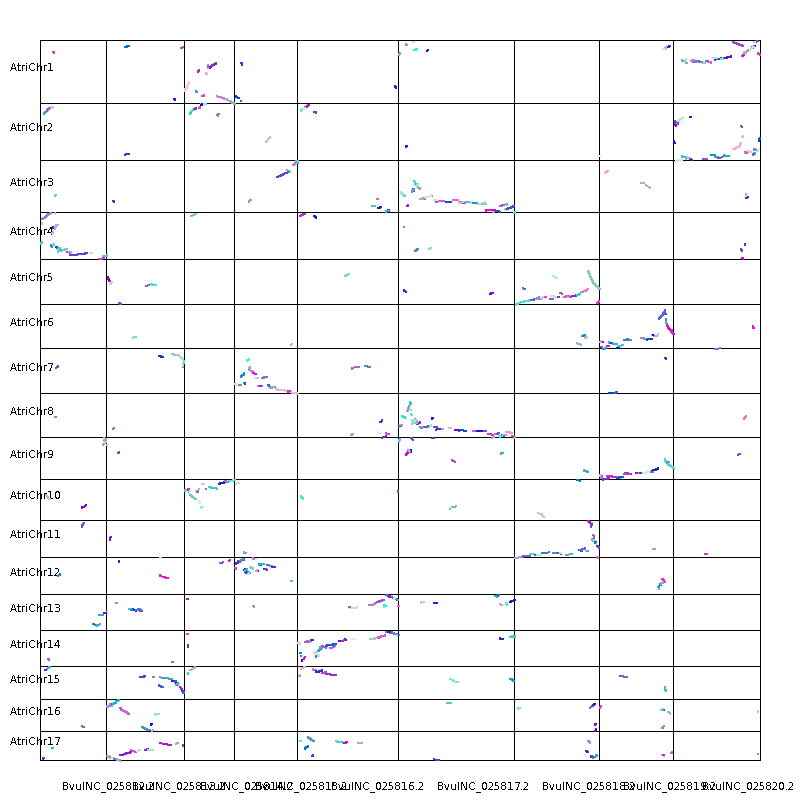


Figure S9. Dot plot of pairwise synteny blocks for plants in Amaranthaceae. (a) Dot plot of collinearity genes between *A. tricolor* and *A. cruentus*. (b) Dot plot of collinearity genes between *A. tricolor* and *A. palmeri*. (c) Dot plot of collinearity genes between *A. tricolor* and *A. hyponchondricus*. (d) Dot plot of collinearity genes between *A. tricolor* and *B. vulgaris*. From these plots, we can confirm that *A. tricolor* had a whole genome duplication event after divergence from *B. vulgaris*.

Figure S10. Alignment between chromosome genomes of *A. cruentus* and *A. tricolor*. The x-axis is 17 chromosomes of *A. tricolor* and y-axis is 17 chromosomes of *A. cruentus*. Large part of pair chromosomes have good collinearity. We used minimap2 with parameters “-ax asm10” to align two genomes and dotPlotly (https://github.com/tpoorten/dotPlotly) with parameters “-l -x -q 0 -m 0” to draw the figure.

Figure S11. Alignment between chromosome genomes of *A. hyponchondricus* and *A. tricolor*. The x-axis is 17 chromosomes of *A. tricolor* and y-axis is 16 chromosomes of *A. hyponchondricus*. Large part of pair chromosomes have good collinearity. We used minimap2 with parameters “-ax asm10” to align two genomes and dotPlotly (https://github.com/tpoorten/dotPlotly) with parameters “-l -x -q 0 -m 0” to draw the figure.

Figure S12. Alignment between chromosome genomes of *A. palmeri* and *A. tricolor*. The x-axis is 17 chromosomes of *A. tricolor* and y-axis is 17 chromosomes of *A. palmeri*. Large part of pair chromosomes have good collinearity. We used minimap2 with parameters “-ax asm10” to align two genomes and dotPlotly (https://github.com/tpoorten/dotPlotly) with parameters “-l -x -q 0 -m 0” to draw the figure.

(c)

(b)

(a)


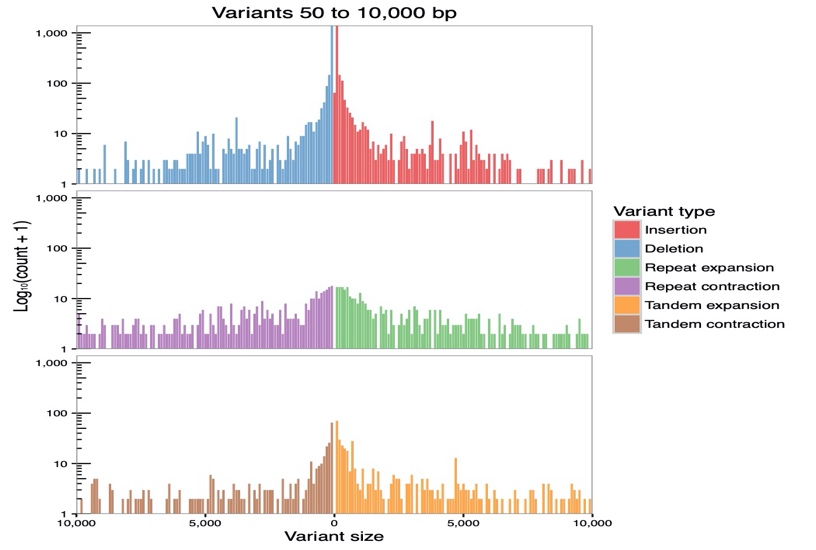


Figure S13. Distribution of structure variations across different sizes between cv. Red and cv. Green. (a) Distribution of variants with size between 50-500 bp. (b) Distribution of variants with size between 500-1000 bp. (c) Distribution of variants with all size. We used nucmer in MUMmer v3.23 to align two genomes and used Assemblytics (Nattestad & Schatz, 2016) to detect variants.

Figure S14. TAD appearances for a 3 Mb region on chromosome 16 at 20 Kb resolution with different parameters. hicFindTADs from HiCExplorer was used to call TADs with parameters “--thresholdComparisons 0.001”, “--thresholdComparisons 0.005”, “--thresholdComparisons 0.01” and “--thresholdComparisons 0.05”, respectively. One of TADs in this region includes key genes of betalain biosynthesis (DODAα1 and CYP76ADα1) and is highlighted in cyan.

Figure S15. A / B compartments of chromosomes for *A. tricolor* with bin size 500 Kb. We used hicPlotMatrix from HiCExplorer to plot pearson correlation of Hi-C matrix. The A / B compartment are classified by gene number, expression level of genes, Hi-C reads count and methylation level.

(b)

(a)


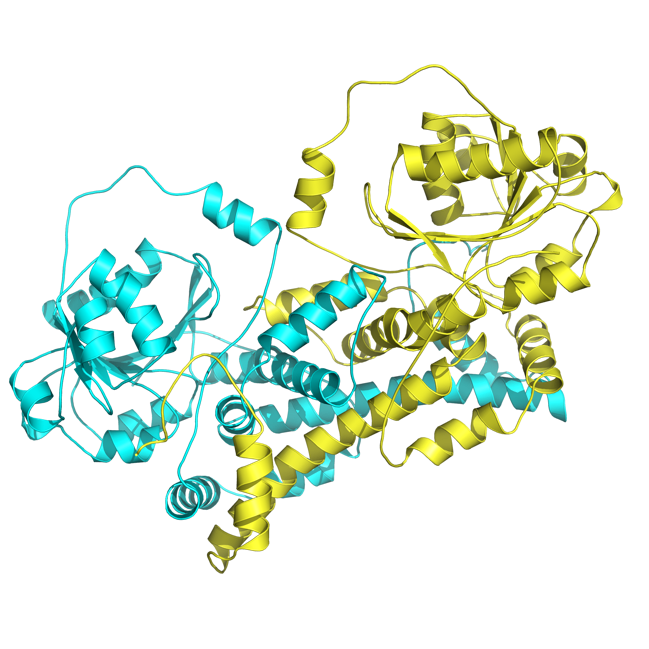


(c)

Figure S16. Evolution, expression and 3D structure of *ADH* genes. (a) Phylogenetic tree of *ADH* genes in Caryophyllales. Clade α and clade β of ADH genes in Amaranthaceae are highlighted in yellow and magenta. A. tricolor has three *ADH* genes, including g5475 (ADHα1), g3135 (ADHβ1) and g7459 (ADHβ2). (b) Expression of *ADH* genes of *A. tricolor* from transcriptome. The expression levels are defined by TPM. (c) 3D structure of ADHα1. The structure is a homodimer and two sections are in yellow and cyan.


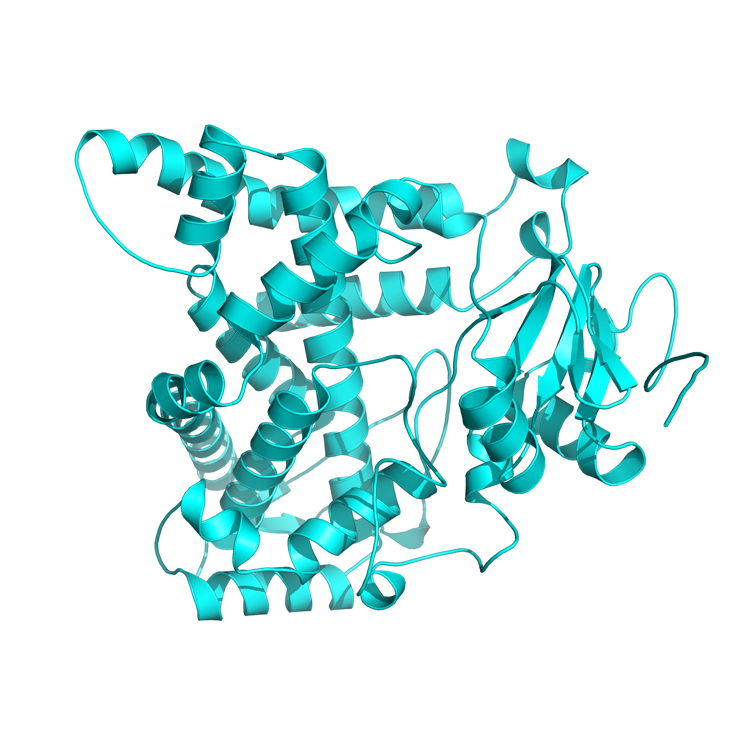


(b)

(a)

Figure S17. Evolution and 3D structure of *CYP76AD* genes. (a) Phylogenetic tree of *CYP76AD* genes in Caryophyllales. This tree is constructed by three paralogous lineages: CYP76AD α, β and γ. (b) 3D structure of CYP76ADα1.

(a)


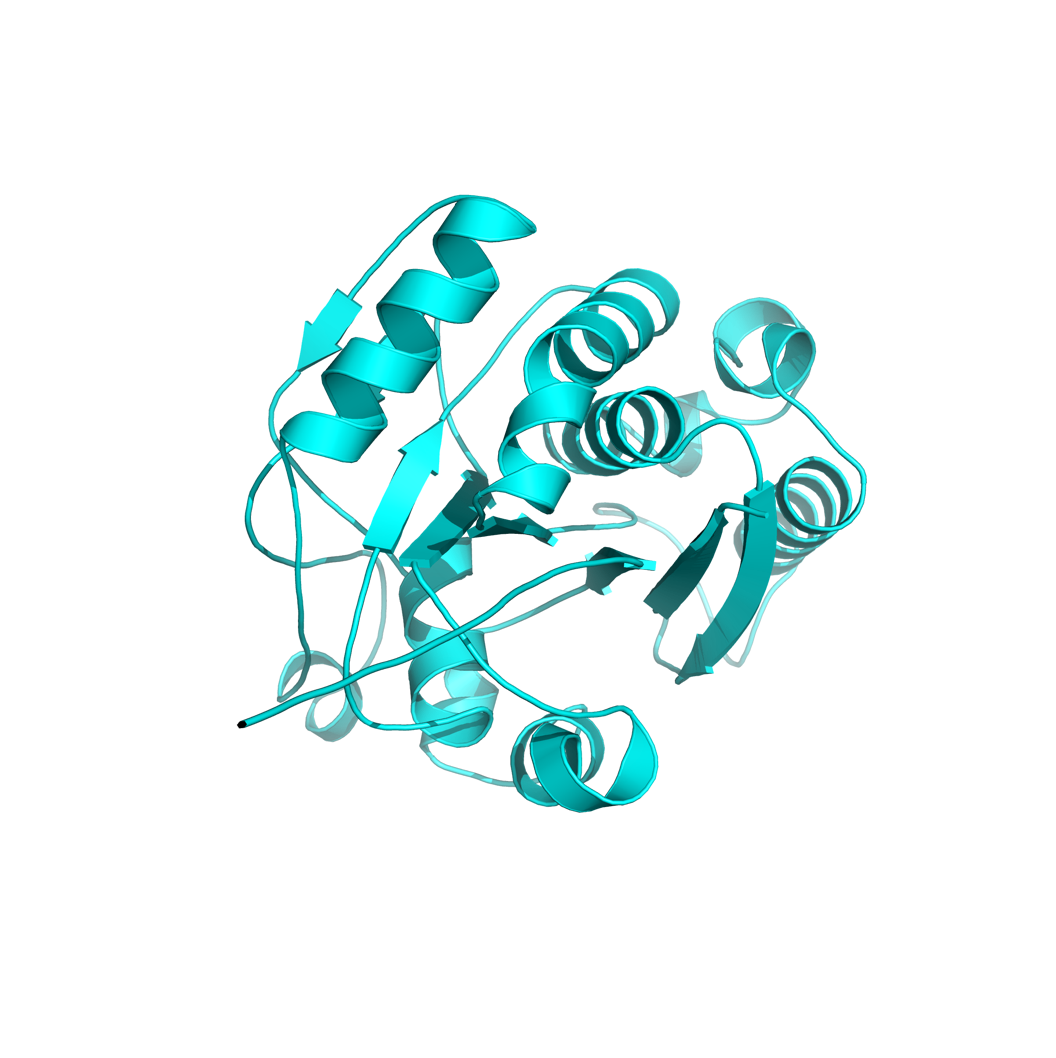


(b)

Figure S18. Evolution and 3D structure of *DODA* genes. (a) Phylogenetic tree of *DODA* genes in Caryophyllales. This tree is constructed by two paralogous lineages: DODA α and β, and *A. tricolor* has three DODA genes, including g14870.t1 (DODAβ1), g14871.t1 (DODAα2), g23453.t1 (DODAα1). (b) 3D structure of DODAα1 (g23453).

(a)


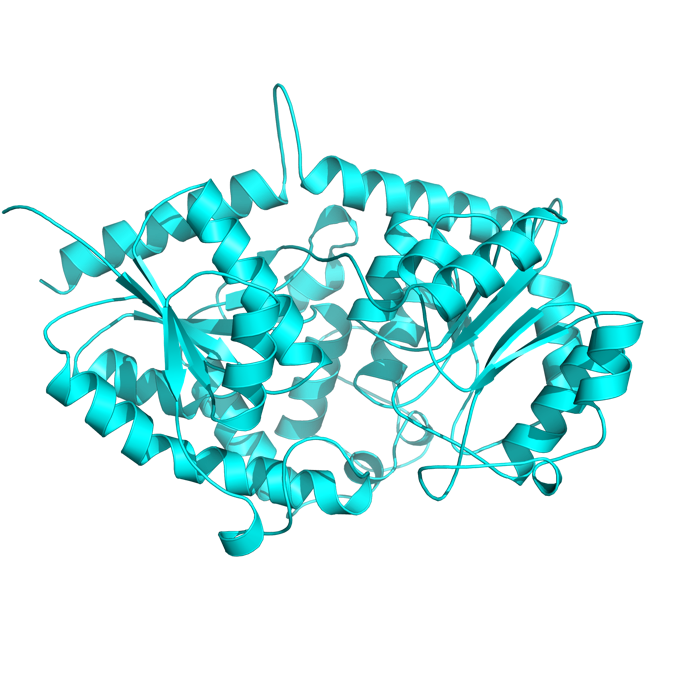


(c)

(b)

Figure S19. Evolution, expression and 3D structure of *cDOPA5GT* gene. (a) Phylogenetic tree of *cDOPA5GT* genes in Caryophyllales. *A. tricolor* has only one *cDOPA5GT* gene. (b) Expression of *cDOPA5GT* gene of *A. tricolor* from transcriptome. The expression levels are defined by TPM. (c) 3D structure of cDOPA5GT.

(a)


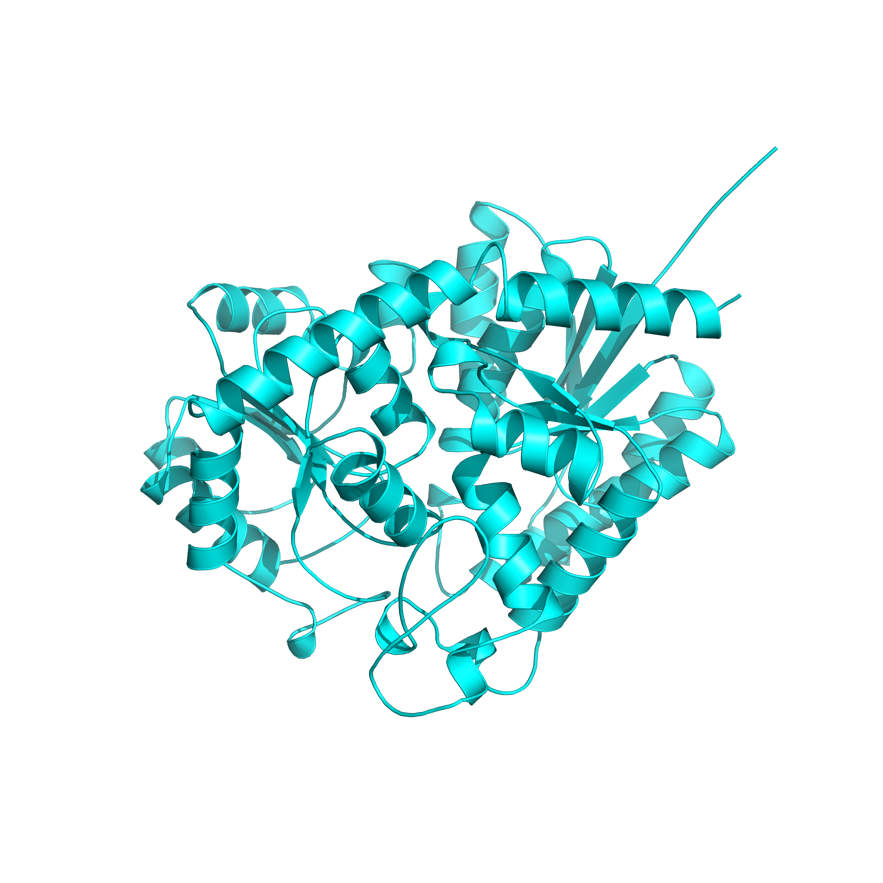


(b)

(c)

Figure S20. Evolution, expression and 3D structure of *B5GT* genes. (a) Phylogenetic tree of *B5GT* genes in Caryophyllales. *A. tricolor* has only six *B5GT* genes. (b) Expression of *B5GT* genes of *A. tricolor* from transcriptome. The expression levels are defined by TPM. (c) 3D structure of B5GT (g8640).

(b)

(a)


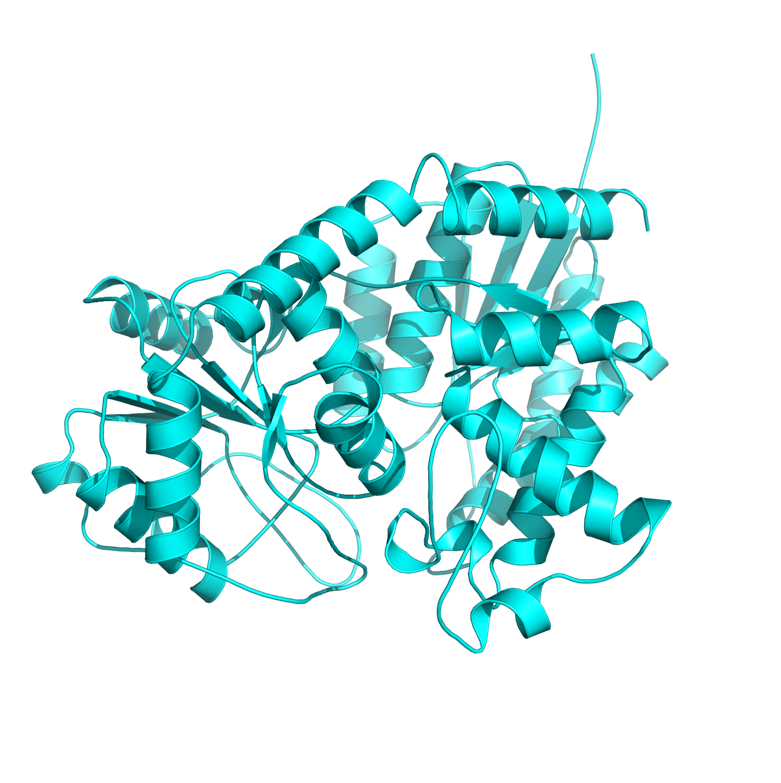


(c)

Figure S21. Evolution, expression and 3D structure of *B6GT* genes. (a) Phylogenetic tree of *B6GT* genes in Caryophyllales. *A. tricolor* has only five *B6GT* genes. (b) Expression of *B6GT* genes of *A. tricolor* from transcriptome. The expression levels are defined by TPM. (c) 3D structure of B6GT (g12720).

(a)


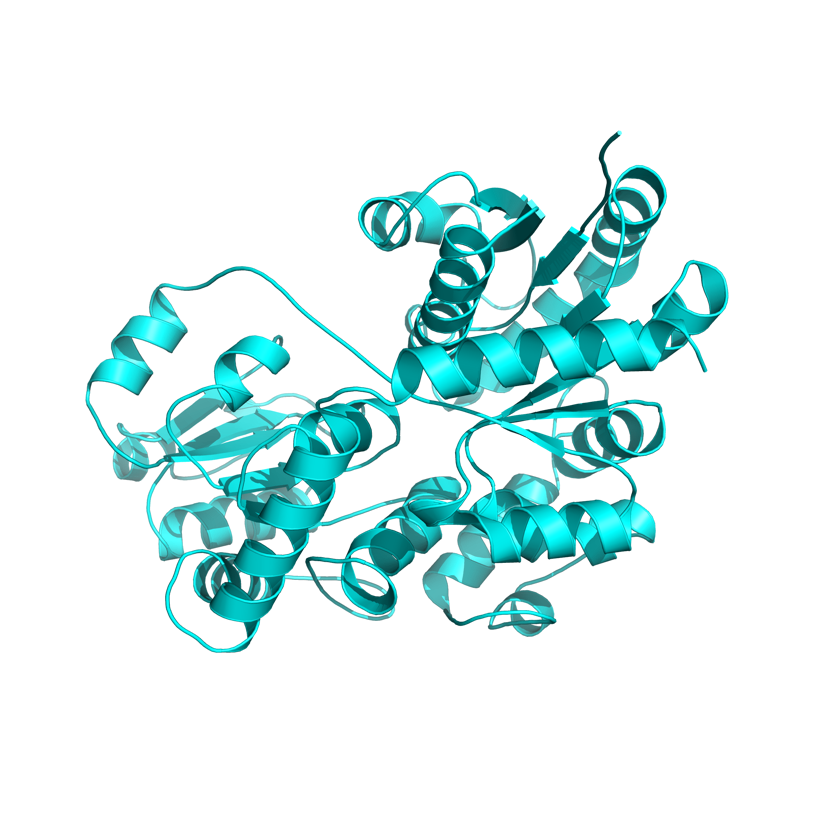


(c)

(b)

Figure S22. Evolution, expression and 3D structure of *UDPGT* genes. (a) Phylogenetic tree of *UDPGT* genes in Caryophyllales. *A. tricolor* has three *UDPGT* genes. (b) Expression of *UDPGT* genes of *A. tricolor* from transcriptome. The expression levels are defined by TPM. (c) 3D structure of UDPGT (g17491).

(a)


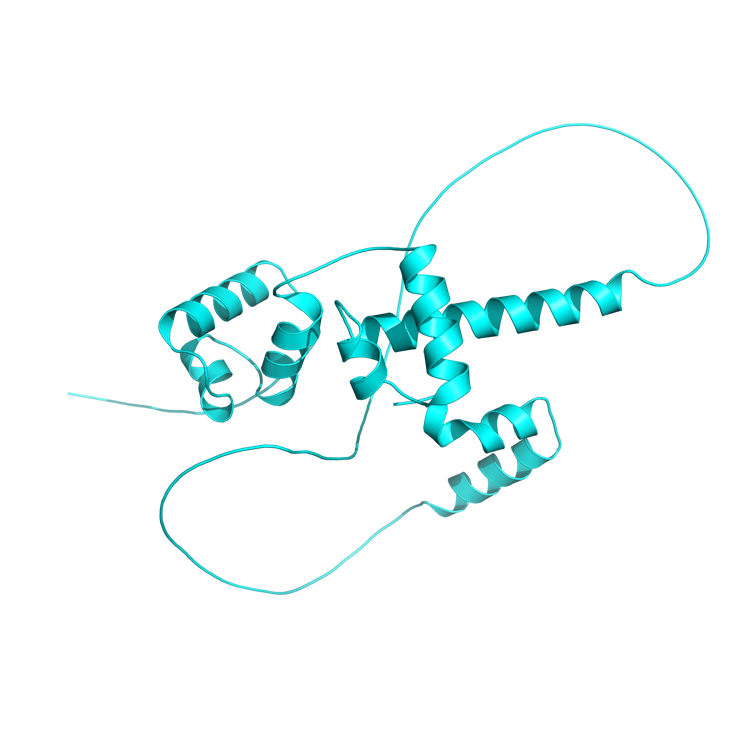


(b)

Figure S23. Evolution and 3D structure of *MYB* genes. (a) Phylogenetic tree of *MYB* genes in Caryophyllales. *A. tricolor* has only two *MYB* genes. (b) 3D structure of MYB (g16810).


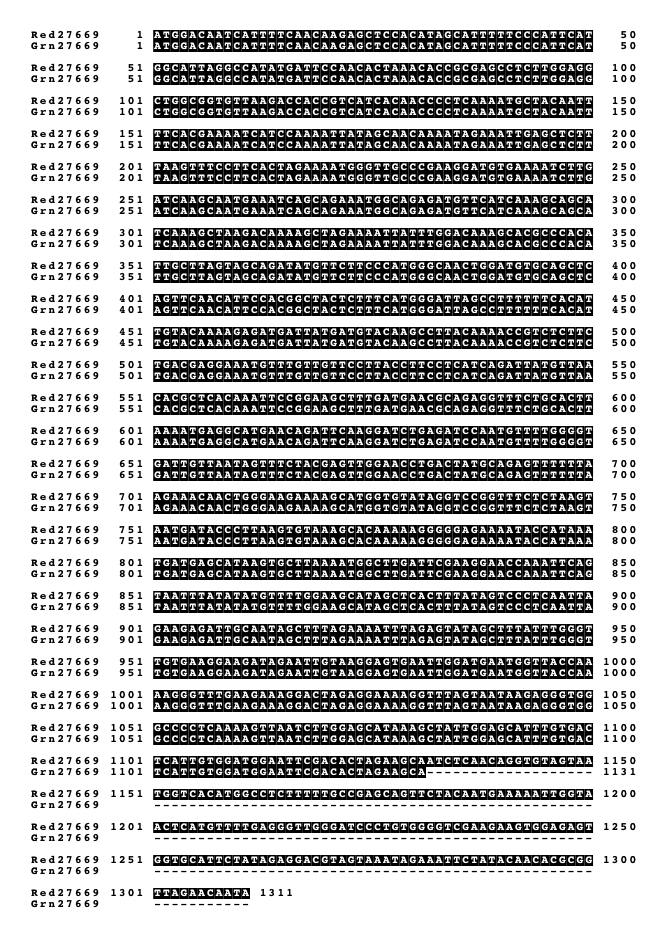


Figure S24. Sequence alignment of two genes (g27669) from two cultivars of A. tricolor. pyBoxshade (https://github.com/mdbaron42/pyBoxshade) was used to show this alignment.

**Supplementary Tables**

Table S1. Sequencing statistics of *A. tricolor*.

| Cultivar | Source | Libraries | Sequence Type | Insert Size | N50 Read Length | Total Raw Data (Gb) | Accession |
| --- | --- | --- | --- | --- | --- | --- | --- |
| cv. Red | Genomic DNA | Illumina | PE | 500 bp | 150 bp | 23.3 | SRR21968682 |
|  |  | Illumina Hi-C | PE | 500 bp | 150 bp | 78.6 | SRR21969976, SRR21969977 |
|  |  | PacBio-HiFi | SE | 15 kb | 13.8 kb | 28.1 | SRR21969943 |
|  |  | Nanopore Ultra-long | SE | 50 kb | 48.4 kb | 52.0 | SRR21970862 |
|  | Transcriptome Sequencing | Illumina | PE | 400 bp | 150 bp | 72.1 | SRR21969979-SRR21969990 |
|  |  | PacBio Iso-Seq | SE | 0.5 ~ 6 kb | 2.1 kb | 1.5 | SRR21970049 |
| cv. Green | Genomic DNA | Illumina | PE | 400 bp | 150 bp | 33.1 | SRR21968681 |
|  |  | PacBio-HiFi | SE | 15 kb | 14.4 kb | 25.2 | SRR21969944 |
|  | Transcriptome Sequencing | Illumina | PE | 400 bp | 150 bp | 25.8 | SRR21970002-SRR21970005 |

Table S2. Statistics of genome assembly for *A. tricolor* cv. Red.

|  | Contigs | | Scaffolds | | Super scaffolds (Chromosomes) | |
| --- | --- | --- | --- | --- | --- | --- |
| Total | 2,544 | 518,713,104 | 2,412 | 519,847,713 | 48 | 520,084,113 |
| Maximum | 1 | 10,322,831 | 1 | 16,114,678 | 1 | 45,445,981 |
| N10 | 6 | 6,800,383 | 3 | 13,432,986 | 1 | 41,158,439 |
| N20 | 15 | 4,397,333 | 8 | 9,559,609 | 2 | 37,339,215 |
| N30 | 30 | 2,701,793 | 14 | 6,841,478 | 3 | 34,495,491 |
| N40 | 55 | 1,596,051 | 25 | 3,480,167 | 5 | 32,177,263 |
| N50 | 98 | 905,879 | 45 | 2,024,549 | 7 | 31,692,477 |
| N60 | 189 | 421,732 | 103 | 511,001 | 8 | 30,081,905 |
| N70 | 353 | 236,359 | 251 | 256,625 | 10 | 27,266,332 |
| N80 | 642 | 138,178 | 526 | 143,750 | 12 | 26,223,628 |
| N90 | 1,171 | 69,518 | 1,045 | 70,114 | 14 | 24,324,110 |

Note: Scaffolds stands for ONT Ultra-long linked scaffold assembly and Super scaffolds stands for chromosome-scale scaffolds anchored by Hi-C.

Table S3. Statistics of each chromosome for *A. tricolor* cv. Red.

| Chromosome name | Length | GC% | Telomere number |
| --- | --- | --- | --- |
| Chr1 | 45,445,981 | 30.57% | 2 |
| Chr2 | 41,158,439 | 32.00% | 2 |
| Chr3 | 37,339,215 | 31.94% | 2 |
| Chr4 | 34,495,491 | 32.20% | 1 |
| Chr5 | 32,224,864 | 32.03% | 2 |
| Chr6 | 32,177,263 | 32.17% | 1 |
| Chr7 | 31,876,255 | 32.22% | 1 |
| Chr8 | 31,692,477 | 34.82% | 1 |
| Chr9 | 30,081,905 | 31.77% | 2 |
| Chr10 | 29,480,995 | 32.40% | 2 |
| Chr11 | 27,266,332 | 30.83% | 2 |
| Chr12 | 26,347,435 | 30.93% | 2 |
| Chr13 | 26,223,628 | 32.21% | 2 |
| Chr14 | 25,584,183 | 32.00% | 2 |
| Chr15 | 24,324,110 | 31.78% | 2 |
| Chr16 | 22,883,222 | 31.21% | 2 |
| Chr17 | 20,436,632 | 32.23% | 2 |

Note: 30 of 34 telomeres are found on the end of chromosomes. Only 4 chromosomes have one telomere on the end.Table S4. Positions of telomeres on the chromosomes of *A. tricolor*.

|  | Start position | End position |
| --- | --- | --- |
| Chr1 | 1 | 9,518 |
| Chr1 | 45,438,939 | 45,445,981 |
| Chr2 | 121 | 7,805 |
| Chr2 | 41,146,628 | 41,158,439 |
| Chr3 | 1 | 7,907 |
| Chr3 | 37,330,933 | 37,339,215 |
| Chr4 | 1 | 12,433 |
| Chr5 | 1 | 6,893 |
| Chr5 | 32,210,540 | 32,224,864 |
| Chr6 | 32,166,299 | 32,177,263 |
| Chr7 | 31,868,894 | 31,876,189 |
| Chr8 | 31,686,343 | 31,692,477 |
| Chr9 | 1 | 9,049 |
| Chr9 | 30,072,364 | 30,081,905 |
| Chr10 | 1 | 12,012 |
| Chr10 | 26,554,441 | 26,564,949 |
| Chr11 | 1 | 11,386 |
| Chr11 | 27,255,491 | 27,266,332 |
| Chr12 | 1 | 6,630 |
| Chr12 | 26,344,513 | 26,347,435 |
| Chr13 | 1 | 16,492 |
| Chr13 | 26,217,858 | 26,223,628 |
| Chr14 | 1 | 14,831 |
| Chr14 | 25,572,073 | 25,584,183 |
| Chr15 | 1 | 14,746 |
| Chr15 | 24,309,155 | 24,324,110 |
| Chr16 | 1 | 9,239 |
| Chr16 | 22,877,721 | 22,883,222 |
| Chr17 | 1 | 9,022 |
| Chr17 | 20,423,101 | 20,436,632 |

Table S5. Positions of centromeres on the chromosomes of *A. tricolor*.

|  | Start position | End position |
| --- | --- | --- |
| Chr1 | 27,597,363 | 29,341,950 |
| Chr2 | 17,404,922 | 19,693,955 |
| Chr3 | 11,581,519 | 12,984,859 |
| Chr4 | 5,470,372 | 7,451,487 |
| Chr5 | 31,652 | 3,154,397 |
| Chr6 | 2,738,277 | 3,656,614 |
| Chr6 | 960,623 | 2,653,948 |
| Chr7 | 1,169,067 | 2,444,851 |
| Chr8 | 12,860,927 | 14,187,427 |
| Chr9 | 12,774,640 | 13,705,402 |
| Chr9 | 6,186,805 | 7,288,010 |
| Chr10 | 28,119,194 | 28,928,902 |
| Chr11 | 8,727,805 | 9,859,124 |
| Chr12 | 23,948,832 | 25,133,504 |
| Chr13 | 24,167,331 | 26,170,617 |
| Chr14 | 22,867,480 | 24,872,289 |
| Chr15 | 20,869,523 | 21,629,810 |
| Chr16 | 13,718,966 | 15,862,534 |
| Chr17 | 271,750 | 3,215,932 |

Table S6. BUSCO assessment of genome assembly for *A. tricolor*.

| BUSCO settings | Augustus using arabidopsis as species | Augustus using coyote_tobacco as species | metaeuk |
| --- | --- | --- | --- |
| Complete BUSCOs | 1,569 | 1,574 | 1,579 |
| Complete ratio | 97.2% | 97.5% | 97.8% |
| Complete and single-copy BUSCOs | 1,523 | 1,527 | 1,537 |
| Complete and single-copy ratio | 94.4% | 94.6% | 95.2% |
| Complete and duplicated BUSCOs | 46 | 47 | 42 |
| Complete and duplicated ratio | 2.9% | 2.9% | 2.6% |
| Fragmented BUSCOs | 14 | 9 | 12 |
| Fragmented ratio | 0.9% | 0.6% | 0.7% |
| Missing BUSCOs | 31 | 31 | 23 |
| Missing ratio | 1.9% | 1.9% | 1.4% |
| Total BUSCO groups searched | 1,614 | 1,614 | 1,614 |

Note: We run BUSCO with different settings to assess our genome.

Table S7. Statistics of gene prediction evidence from full-length cDNA mapping.

|  | *A. tricolor* |
| --- | --- |
| CCS reads number | 1,157,088 |
| Iso-seq3 non-redundant isoforms | 144,337 |
| Ratio of non-redundant isoforms | 12.47% |
| Gmap+blat2hints.pl hits | 134,627 |
| Ratio in total | 11.63% |
| Hints summary | |
| Total exon part hints | 249,671 |
| Total exon hints | 677,852 |
| Total intron hints | 793,689 |

Table S8. Statistics of gene prediction evidence from homology alignment.

| Species | A. tricolor |
| --- | --- |
| *A.* *thaliana* | 25,891 |
| *A. hypochondriacus* | 30,548 |
| *C. quinoa* | 47,342 |
| *S. oleracea* | 30,905 |
| *B. vulgaris* | 32,932 |
| Swiss plant | 44,350 |
| Hints summary | |
| Total CDSpart hints | 1,052,608 |
| Total intron hints | 835,926 |
| Total start hints | 116,933 |

Table S9. Statistics of gene prediction evidence from RNA-seq mapping.

|  | *A. tricolor* |
| --- | --- |
| Number of reads | 315,787,984 |
| Average read length | 300 |
| Uniquely mapped reads number | 288,800,773 |
| Uniquely mapped reads % | 91.45% |
| Number of reads mapped to multiple loci | 14,991,227 |
| % of reads mapped to multiple loci | 4.75% |
| Hints summary | |
| Total intron hints | 435,738 |

Table S10. Gene statistics of related plant species.

| **Species Name** | **Gene number reported** | **Gene number used here** | **Gene number used %** | **Non-intact gene number** | **Total CDS length** | **Coding ratio in assembly** | **Avg CDS length (bp)** | **Avg exon（CDS） number** | **Genome length** |
| --- | --- | --- | --- | --- | --- | --- | --- | --- | --- |
| *A. tricolor* | 27,813 | 27,813 | 100% | 0 | 34,660,456 | 6.66% | 1,246 | 5.30 | 520,084,113 |
| *A. tuberculatus* | 30,771 | 44,093 | 143% | -13,322 | 38,843,670 | 5.64% | 863 | 3.15 | 688,987,999 |
| *A. palmeri* | 26,506 | 26,506 | 100% | 0 | 30,592,772 | 7.43% | 1,154 | 5.07 | 411,927,395 |
| *A. cruentus* | 25,477 | 25,248 | 99% | 229 | 28,807,456 | 7.77% | 1,140 | 4.85 | 370,913,848 |
| *A. hypochondriacus* | 23,847 | 23,641 | 99% | 206 | 25,302,267 | 6.39% | 1,068 | 4.87 | 395,806,076 |
| *A. hybridus* | 24,325 | 23,820 | 98% | 505 | 27,977,977 | 6.79% | 1,174 | 4.79 | 411,833,878 |
| *B. vulgaris* | 32,874 | 24,351 | 74% | 8,523 | 32,037,164 | 5.65% | 1,315 | 4.92 | 566,550,431 |
| *C. quinoa* | 63,173 | 49,138 | 78% | 14,035 | 62,909,207 | 4.72% | 1,280 | 4.91 | 1,333,551,035 |
| *S. oleracea* | 25,495 | 25,609 | 100% | -114 | 33,518,151 | 3.85% | 1,308 | 4.90 | 869,946,296 |
| *H. undatus* | 27,735 | 27,735 | 100% | 0 | 32,152,977 | 2.32% | 1,159 | 5.06 | 1,387,322,347 |
| *S. chinensis* | 23,490 | 23,490 | 100% | 0 | 26,742,763 | 3.02% | 1,138 | 5.01 | 886,727,160 |
| *D. caryophyllus* | 43,266 | 43,268 | 100% | -2 | 48,420,457 | 8.51% | 1,119 | 4.74 | 568,887,315 |
| *A. vesiculosa* | 25,123 | 25,123 | 100% | 0 | 32,871,522 | 7.82% | 1,308 | 5.84 | 420,285,881 |
| *F. tataricum* | 33,366 | 36,613 | 110% | -3,247 | 36,487,884 | 8.04% | 996 | 4.30 | 453,931,790 |
| *A. thaliana* | - | 27,444 | - | - | 33,519,686 | 28.01% | 1,221 | 5.15 | 119,668,634 |

Table S11. Statistics of functional annotation for genes in *A. tricolor* based on different databases*.*

|  | A. tricolor |
| --- | --- |
| Total gene number | 27,813 |
| NR hit | 24,717 |
| NR hit % | 88.87% |
| KEGG hit | 19,393 |
| KEGG hit % | 69.73% |
| Uniprot (Swiss-Prot) hit | 19,619 |
| Uniprot (Swiss-Prot) hit % | 70.54% |
| InterPro hits | 25,313 |
| InterPro hits % | 91.01% |
| InterPro term | 21,771 |
| InterPro term % | 78.28% |
| GO terms | 16,161 |
| GO terms % | 58.11% |
| All function | 25,820 |
| All function % | 92.83% |

Table S12. The summary of annotated tRNAs in *A. tricolor*.

| tRNA name | Total number | Anti-codon: number |
| --- | --- | --- |
| tRAN-Ala | 29 | AGC:13, GGC:1, CGC:3, TGC:12 |
| tRAN-Arg | 79 | ACG:40, GCG:2, CCG:4, TCG:6, CCT:6, TCT:21 |
| tRAN-Asn | 186 | ATT:102, GTT:84 |
| tRAN-Asp | 37 | ATC:1, GTC:36 |
| tRAN-Cys | 24 | ACA:1, GCA:23 |
| tRAN-Gln | 32 | CTG:6, TTG:26 |
| tRAN-Glu | 36 | CTC:12, TTC:24 |
| tRAN-Gly | 49 | ACC:1, GCC:32, CCC:4, TCC:12 |
| tRAN-His | 77 | ATG:5, GTG:72 |
| tRAN-Ile | 206 | AAT:198, TAT:8 |
| tRAN-iMet | 7 | CAT:7 |
| tRAN-Leu | 82 | AAG:11, CAG:3, TAG:19, CAA:41, TAA:8 |
| tRAN-Lys | 47 | CTT:22, TTT:25 |
| tRAN-Met | 54 | CAT:54 |
| tRAN-Phe | 60 | AAA:20, GAA:40 |
| tRAN-Pro | 51 | AGG:10, GGG:1, CGG:3, TGG:37 |
| tRAN-Ser | 66 | AGA:18, GGA:5, CGA:5, TGA:17, GCT:21 |
| tRAN-Thr | 42 | AGT:14, GGT:6, CGT:2, TGT:20 |
| tRAN-Trp | 17 | CCA:17 |
| tRAN-Tyr | 56 | ATA:3, GTA:53 |
| tRAN-Val | 48 | AAC:12, GAC:24, CAC:6, TAC:6 |
| tRAN-Sup | 4 | TTA:1, TCA:3 |
| Total | 1,289 |  |

Note: tRNA-Sup is suppressor tRNA. tRNA-iMet is initial tRNA-Met.

Table S13. The summary of annotated rRNAs and other ncRNAs in *A. tricolor*.

|  | Number |
| --- | --- |
| rRNAs | |
| 5S rRNA | 2,840 |
| 5.8S rRNA | 414 |
| 18S rRNA | 426 |
| 28S rRNA | 433 |
| Total rRNA | 4,113 |
| Other ncRNAs | |
| LSU rRNA eukarya | 447 |
| SSU rRNA eukarya | 443 |
| U5 | 267 |
| Intron_gpII | 231 |
| snoR71 | 143 |
| MIR811 | 51 |
| U1 | 44 |
| U2 | 30 |
| U6 | 22 |
| U11 | 20 |
| MIR169_2 | 14 |
| MIR159 | 11 |
| snoZ159 | 11 |
| IsrR | 10 |
| Others | 331 |
| Total other ncRNAs | 2,075 |

Table S14. Statistics of annotated transposable elements in *A. tricolor*.

|  | *A. tricolor* | |
| --- | --- | --- |
| TE class | length | percent |
| DNA | 192,159,999 | 36.95% |
| LTR | 147,817,623 | 28.42% |
| MITE | 6,988,608 | 1.34% |
| LINE | 6,180,647 | 1.19% |
| SINE | 589,487 | 0.11% |
| RC | 56,644 | 0.01% |

Table S15. Statistics of annotated transcription factors in *A. tricolor*.

| TF Family | Number | TF Family | Number |
| --- | --- | --- | --- |
| AP2 | 18 | LBD | 35 |
| ARF | 23 | LFY | 2 |
| ARR-B | 11 | LSD | 5 |
| B3 | 30 | MIKC_MADS | 27 |
| BBR-BPC | 6 | M-type_MADS | 21 |
| BES1 | 6 | MYB | 85 |
| bHLH | 134 | MYB_related | 60 |
| bZIP | 62 | NAC | 68 |
| C2H2 | 84 | NF-X1 | 2 |
| C3H | 53 | NF-YA | 7 |
| CAMTA | 6 | NF-YB | 11 |
| CO-like | 10 | NF-YC | 6 |
| CPP | 8 | Nin-like | 12 |
| DBB | 7 | RAV | 4 |
| Dof | 31 | S1Fa-like | 2 |
| E2F/DP | 6 | SAP | 2 |
| EIL | 4 | SBP | 17 |
| ERF | 93 | SRS | 6 |
| FAR1 | 31 | STAT | 1 |
| G2-like | 37 | TALE | 18 |
| GATA | 25 | TCP | 20 |
| GeBP | 4 | Trihelix | 31 |
| GRAS | 34 | VOZ | 2 |
| GRF | 10 | Whirly | 3 |
| HB-other | 8 | WOX | 10 |
| HB-PHD | 2 | WRKY | 62 |
| HD-ZIP | 35 | YABBY | 6 |
| HRT-like | 1 | ZF-HD | 12 |
| HSF | 21 |  |  |
| Total | | 1,337 | |

Note: The TFs are annotated by PlantTFDB (<http://planttfdb.gao-lab.org/prediction.php>).

Table S16. Statistics of annotated plant disease resistance genes in *A. tricolor*.

| Class of resistance gene | Number |
| --- | --- |
| CK | 76 |
| CL | 13 |
| CLK | 4 |
| CN | 56 |
| CNL | 39 |
| CT | 1 |
| KIN | 619 |
| L | 24 |
| N | 70 |
| NK | 1 |
| NL | 41 |
| NLK | 1 |
| RLK | 183 |
| RLP | 105 |
| T | 4 |
| TNL | 1 |
| TRAN | 2 |
| Total | 1,240 |

Note: The annotation was done by DRAGO2-API (Osuna-Cruz *et al.*, 2018) (<https://github.com/sequentiabiotech/DRAGO2-API>) based on <http://prgdb.org/prgdb4/drago3>.

Table S17. Statistics of tandem repeats for plants in Caryophyllales.

| **Species Name** | Tandem repeats length | Tandem repeats percent | Genome length | Tandem repeats N50 length | max length | median length |
| --- | --- | --- | --- | --- | --- | --- |
| *A. tricolor* | 119,118,175 | 22.90% | 520,084,113 | 60,193 | 3,122,746 | 69 |
| *A. tuberculatus* | 37,473,345 | 5.44% | 688,987,999 | 242 | 60,154 | 65 |
| *A. palmeri* | 27,212,791 | 6.61% | 411,927,395 | 507 | 163,575 | 64 |
| *A. cruentus* | 14,436,972 | 3.89% | 370,913,848 | 131 | 13,585 | 60 |
| *A. hypochondriacus* | 19,963,046 | 5.04% | 395,806,076 | 209 | 32,653 | 61 |
| *A. hybridus* | 22,057,112 | 5.36% | 411,833,878 | 330 | 173,157 | 62 |
| *B. vulgaris* | 26,737,606 | 4.72% | 566,550,431 | 206 | 23,572 | 66 |
| *C. quinoa* | 145,050,484 | 10.88% | 1,333,551,035 | 22,570 | 363,256 | 64 |
| *S. oleracea* | 58,987,065 | 6.78% | 869,946,296 | 148 | 40,269 | 96 |
| *H. undatus* | 144,559,755 | 10.42% | 1,387,322,347 | 1,493 | 444,807 | 66 |
| *S. chinensis* | 75,574,678 | 8.52% | 886,727,160 | 59,131 | 2,103,632 | 66 |
| *D. caryophyllus* | 22,643,705 | 3.98% | 568,887,315 | 156 | 14,286 | 56 |
| *A. vesiculosa* | 8,508,285 | 2.02% | 420,285,881 | 154 | 52,769 | 53 |
| *F. tataricum* | 16,175,286 | 3.56% | 453,931,790 | 171 | 37,806 | 62 |

Table S18. Statistics of closely related genomes of Caryophyllales and *Arabidopsis thaliana*.

| **Species Name** | **Family / Subfamily** | **Ploidy** | **Sequencing technology** | **Genome Size (G)** | **Assembly size (G)** | **GC percent** | **Contig N50 size (M)** |
| --- | --- | --- | --- | --- | --- | --- | --- |
| *A. tricolor* (Chinese spinach) | Amaranthaceae / Amaranthoideae | 2n = 34 | PacBio CCS, ONT, HiC | 0.63 | 0.52 | 31.93% | 0.91 |
| *A. tuberculatus* | Amaranthaceae / Amaranthoideae | 2n = 32 | PacBio | 0.68 | 0.69 | 34.94% | 1.74 |
| *A. palmeri* | Amaranthaceae / Amaranthoideae | 2n = 32 | PacBio, HiC | 0.42 | 0.41 | 33.18% | 2.54 |
| *A. cruentus* | Amaranthaceae / Amaranthoideae | 2n = 34 | ONT, HiC | 0.40 | 0.37 | 33.08% | 1.02 |
| *A. hypochondriacus* | Amaranthaceae / Amaranthoideae | 2n = 32 | PacBio, HiC | 0.47 | 0.40 | 32.71% | 1.15 |
| *A. hybridus* | Amaranthaceae / Amaranthoideae | 2n = 32 | PacBio | 0.50 | 0.41 | 33.01% | 2.26 |
| *B. vulgaris* (sugar beet) | Amaranthaceae / Chenopodioideae | 2n = 18 | Roche/454, Illumina and Sanger | 0.73 | 0.57 | 36.14% | 2 |
| *C. quinoa* | Amaranthaceae / Chenopodioideae | 2n = 36 | PacBio, BioNano | 1.5 | 1.33 | 36.80% | 1.6 |
| *S. oleracea* (spinach) | Amaranthaceae / Chenopodioideae | 2n = 12 | Illumina, BioNano | 1.01 | 0.87 | 37.82% | 0.02 |
| *H. undatus* (pitaya) | Cactaceae | 2n = 22 | PacBio, 10×, Hi-C | 1.58 | 1.41 | 36.90% | 0.58 |
| *S. chinensis* (jojoba) | Simmondsiaceae | 2n = 52 | PacBio, Illumina, Hi-C | 1.03 | 0.89 | 37.28% | 5.2 |
| *D. caryophyllus* (carnation) | Caryophyllaceae | 2n = 30 | Illumina, Roche | 0.62 | 0.57 | 36.36% | 0.02 |
| *A. vesiculosa* (waterwheel) | Droseraceae | 2n = 38 | PacBio, Illumina | 0.51 | 0.42 | 39.69% | 0.31 |
| *F. tataricum* (Tartary buckwheat) | Polygonaceae | 2n = 16 | PacBio, Illumina, Hi-C | 0.54 | 0.49 | 38.09% | 0.55 |
| *A. thaliana* (thale cress) | Brassicaceae | 2n = 10 | - | 0.12 | 0.12 | 36.06% | - |

Table S19. Statistics of AT tandem repeats for plants in Caryophyllales.

| **Species Name** | AT Tandem length | AT Tandem percent | Genome length | N50 length of AT repeat | max length of AT repeat | median length of AT repeat |
| --- | --- | --- | --- | --- | --- | --- |
| *A. tricolor* | 14,503,590 | 2.79% | 520,084,113 | 3,333 | 15,757 | 117 |
| *A. tuberculatus* | 4,646,533 | 0.67% | 688,987,999 | 192 | 1,478 | 134 |
| *A. palmeri* | 4,325,097 | 1.05% | 411,927,395 | 555 | 10,659 | 170 |
| *A. cruentus* | 2,156,025 | 0.58% | 370,913,848 | 205 | 3,275 | 104 |
| *A. hypochondriacus* | 5,460,347 | 1.38% | 395,806,076 | 1,110 | 17,751 | 85 |
| *A. hybridus* | 2,628,032 | 0.64% | 411,833,878 | 271 | 5,088 | 81 |
| *B. vulgaris* | 423,424 | 0.07% | 566,550,431 | 60 | 709 | 47 |
| *C. quinoa* | 1,063,915 | 0.08% | 1,333,551,035 | 74 | 670 | 60 |
| *S. oleracea* | 511,212 | 0.06% | 869,946,296 | 81 | 570 | 62 |
| *H. undatus* | 27,836,812 | 2.01% | 1,387,322,347 | 988 | 14,867 | 95 |
| *S. chinensis* | 410,559 | 0.05% | 886,727,160 | 38 | 254 | 34 |
| *D. caryophyllus* | 105,325 | 0.02% | 568,887,315 | 41 | 636 | 35 |
| *A. vesiculosa* | 61,142 | 0.01% | 420,285,881 | 31 | 114 | 30 |
| *F. tataricum* | 1,269,887 | 0.28% | 453,931,790 | 65 | 878 | 57 |

Table S20. Statistics of contigs with different AT tandem repeat end numbers.

|  | 0 AT repeat end | | 1 AT repeat end | | 2 AT repeat ends | |
| --- | --- | --- | --- | --- | --- | --- |
|  | number | length | number | length | number | length |
| Total | 1,086 | 184,802,206 | 908 | 226,125,624 | 550 | 107,785,274 |
| N10 | 1 | 10,202,138 | 3 | 6,230,812 | 1 | 4,912,409 |
| N20 | 4 | 5,997,186 | 7 | 4,376,288 | 5 | 2,619,271 |
| N30 | 8 | 3,760,893 | 14 | 2,683,486 | 11 | 1,473,968 |
| N40 | 14 | 2,426,354 | 25 | 1,561,937 | 20 | 950,475 |
| N50 | 23 | 1,531,040 | 44 | 901,270 | 36 | 506,366 |
| N60 | 47 | 502,879 | 81 | 453,964 | 62 | 339,536 |
| N70 | 104 | 212,428 | 148 | 265,342 | 105 | 209,972 |
| N80 | 235 | 101,398 | 256 | 168,891 | 167 | 141,023 |
| N90 | 505 | 48,129 | 436 | 95,087 | 274 | 72,961 |

Table S21. Statistics of CCS reads with different AT tandem repeat end numbers.

|  | 0 AT repeat end | | 1 AT repeat end | | 2 AT repeat ends | |
| --- | --- | --- | --- | --- | --- | --- |
|  | number | length | number | length | number | length |
| Total | 1,986,189 | 27,206,272,979 | 62,953 | 845,849,970 | 816 | 10,797,582 |
| N10 | 135,637 | 18,072 | 4,324 | 17,687 | 56 | 17,138 |
| N20 | 294,137 | 16,437 | 9,360 | 16,099 | 122 | 15,711 |
| N30 | 465,649 | 15,354 | 14,803 | 15,053 | 194 | 14,775 |
| N40 | 648,175 | 14,492 | 20,586 | 14,228 | 269 | 14,096 |
| N50 | 840,879 | 13,766 | 26,686 | 13,529 | 347 | 13,433 |
| N60 | 1,043,379 | 13,123 | 33,087 | 12,912 | 429 | 12,932 |
| N70 | 1,255,470 | 12,546 | 39,780 | 12,370 | 515 | 12,331 |
| N80 | 1,477,243 | 11,987 | 46,770 | 11,819 | 604 | 11,898 |
| N90 | 1,710,611 | 11,272 | 54,149 | 11,000 | 697 | 11,189 |

Table S22. Statistics of orthogroups in different plants defined by OrthoFinder.

|  | Number of genes | Number of genes in orthogroups | Number of unassigned genes | Percentage of genes in orthogroups | Percentage of unassigned genes | Number of orthogroups containing species | Percentage of orthogroups containing species | Number of species-specific orthogroups | Number of genes in species-specific orthogroups | Percentage of genes in species-specific orthogroups |
| --- | --- | --- | --- | --- | --- | --- | --- | --- | --- | --- |
| *A. vesiculosa* | 25,123 | 23,923 | 1,200 | 95.2 | 4.8 | 10,868 | 42.7 | 180 | 710 | 2.8 |
| *A. cruentus* | 25,248 | 24,023 | 1,225 | 95.1 | 4.9 | 14,535 | 57.2 | 90 | 234 | 0.9 |
| *A. hybridus* | 24,662 | 22,922 | 1,740 | 92.9 | 7.1 | 13,593 | 53.5 | 116 | 330 | 1.3 |
| *A. hypochondriacus* | 23,677 | 22,869 | 808 | 96.6 | 3.4 | 14,004 | 55.1 | 39 | 96 | 0.4 |
| *A. palmeri* | 26,506 | 24,539 | 1,967 | 92.6 | 7.4 | 13,348 | 52.5 | 446 | 1,228 | 4.6 |
| *A. tricolor* | 27,813 | 25,892 | 1,921 | 93.1 | 6.9 | 14,004 | 55.1 | 197 | 810 | 2.9 |
| *A. tuberculatus* | 44,093 | 41,757 | 2,336 | 94.7 | 5.3 | 15,826 | 62.2 | 843 | 4,021 | 9.1 |
| *A. thaliana* | 27,444 | 25,299 | 2,145 | 92.2 | 7.8 | 12,549 | 49.4 | 766 | 3,753 | 13.7 |
| *B. vulgaris* | 24,351 | 23,682 | 669 | 97.3 | 2.7 | 13,252 | 52.1 | 96 | 368 | 1.5 |
| *C. quinoa* | 49,138 | 48,570 | 568 | 98.8 | 1.2 | 14,080 | 55.4 | 389 | 2,293 | 4.7 |
| *D. caryophyllus* | 43,268 | 40,485 | 2,783 | 93.6 | 6.4 | 12,287 | 48.3 | 1,264 | 10,354 | 23.9 |
| *F. tataricum* | 36,613 | 32,577 | 4,036 | 89 | 11 | 13,591 | 53.4 | 800 | 2,841 | 7.8 |
| *H. undatus* | 27,735 | 25,775 | 1,960 | 92.9 | 7.1 | 13,329 | 52.4 | 317 | 1,015 | 3.7 |
| *S. chinensis* | 23,490 | 21,530 | 1,960 | 91.7 | 8.3 | 12,893 | 50.7 | 339 | 1,508 | 6.4 |
| *S. oleracea* | 25,609 | 25,173 | 436 | 98.3 | 1.7 | 13,442 | 52.9 | 182 | 831 | 3.2 |

Table S23. Summary of classification of duplicated genes by duplicate_gene_classifier from MCScanX.

| Type | | Singleton | Dispersed | Proximal | Tandem | WGD / Segmental | Total |
| --- | --- | --- | --- | --- | --- | --- | --- |
| *A. tricolor* | number | 4,145 | 12,022 | 1,266 | 2,219 | 8,161 | 27,813 |
|  | percent | 14.90% | 43.22% | 4.55% | 7.98% | 29.34% | 100.00% |
| *A. cruentus* | number | 4,230 | 12,147 | 863 | 2,156 | 5,852 | 25,248 |
|  | percent | 16.75% | 48.11% | 3.42% | 8.54% | 23.18% | 100.00% |
| *A. palmeri* | number | 4,713 | 9,900 | 1,100 | 2,164 | 8,629 | 26,506 |
|  | percent | 17.78% | 37.35% | 4.15% | 8.16% | 32.55% | 100.00% |
| *B. vulgaris* | number | 3,088 | 13,795 | 1,085 | 2,612 | 3,771 | 24,351 |
|  | percent | 12.68% | 56.65% | 4.46% | 10.73% | 15.49% | 100.00% |
| *S. oleracea* | number | 2,814 | 19,075 | 923 | 2,567 | 230 | 25,609 |
|  | percent | 10.99% | 74.49% | 3.60% | 10.02% | 0.90% | 100.00% |
| *C. quina* | number | 581 | 15,871 | 1,866 | 4,127 | 26,693 | 49,138 |
|  | percent | 1.18% | 32.30% | 3.80% | 8.40% | 54.32% | 100.00% |
| *D. caryophyllus* | number | 3,797 | 32,195 | 1,425 | 3,905 | 1,946 | 43,268 |
|  | percent | 8.78% | 74.41% | 3.29% | 9.03% | 4.50% | 100.00% |
| *H. undatus* | number | 3,465 | 10,764 | 1,676 | 2,980 | 8,850 | 27,735 |
|  | percent | 12.49% | 38.81% | 6.04% | 10.74% | 31.91% | 100.00% |
| *S. chinensis* | number | 4,218 | 11,570 | 1,340 | 1,959 | 4,403 | 23,490 |
|  | percent | 17.96% | 49.26% | 5.70% | 8.34% | 18.74% | 100.00% |
| *A. vesiculosa* | number | 1,901 | 10,627 | 399 | 591 | 11,605 | 25,123 |
|  | percent | 7.57% | 42.30% | 1.59% | 2.35% | 46.19% | 100.00% |
| *F. tataricum* | number | 5,325 | 16,849 | 1,902 | 3,549 | 8,988 | 36,613 |
|  | percent | 14.54% | 46.02% | 5.19% | 9.69% | 24.55% | 100.00% |
| *A. thaliana* | number | 3,750 | 10,782 | 1,087 | 3,351 | 8,474 | 27,444 |
|  | percent | 13.66% | 39.29% | 3.96% | 12.21% | 30.88% | 100.00% |

Table S24. Statistics of genome assembly for *A. tricolor* cv. Green.

|  | Contigs | |
| --- | --- | --- |
| Total | 3,334 | 555,444,195 |
| Maximum | 1 | 12,077,820 |
| N10 | 6 | 6,243,764 |
| N20 | 18 | 3,712,391 |
| N30 | 36 | 2,530,314 |
| N40 | 67 | 1,279,399 |
| N50 | 132 | 569,927 |
| N60 | 264 | 316,668 |
| N70 | 503 | 178,415 |
| N80 | 907 | 107,733 |
| N90 | 1,637 | 54,005 |
| Minimum | 1 | 8,968 |

Table S25. Statistics of structure variations between *A. tricolor* cv. Red and cv. Green.

|  | Size range | 50-500 bp | 500-10000 bp | Total |
| --- | --- | --- | --- | --- |
| Insertion | Count | 1,779 | 345 | 2,124 |
|  | Total bp | 193,989 | 1,000,806 | 1,194,795 |
| Deletion | Count | 1,675 | 331 | 2,006 |
|  | Total bp | 181,747 | 1,058,522 | 1,240,269 |
| Tandem expansion | Count | 145 | 217 | 362 |
|  | Total bp | 29,521 | 739,900 | 769,421 |
| Tandem contraction | Count | 125 | 147 | 272 |
|  | Total bp | 23,414 | 560,140 | 583,554 |
| Repeat expansion | Count | 73 | 245 | 318 |
|  | Total bp | 21,017 | 852,219 | 873,236 |
| Repeat contraction | Count | 69 | 267 | 336 |
|  | Total bp | 18,195 | 1,035,013 | 1,053,208 |

Note: Total for all structural variants: 5,418, totaling 5,714,483 bp.

Table S26. Gene ID of betalain biosynthesis in *A. tricolor*.

| Gene | Number | Gene ID |
| --- | --- | --- |
| ADH | 3 | g5475 (ADHα1), g3135 (ADHβ1), g7459 (ADHβ2) |
| CYP76AD | 8 | g23455 (CYP76ADα1), g7779 (CYP76ADβ1), g24899 (CYP76ADβ2), g22247 (CYP76ADβ3), g4847 (CYP76ADβ4), g4848 (CYP76ADβ5), g18040 (CYP76ADγ1), g24301 (CYP76ADγ2) |
| DODA | 3 | g14870 (DODAβ1), g14871 (DODAα2), g23453 (DODAα1) |
| cDOPA5GT | 1 | g691 |
| B5GT | 6 | g22433, g8640, g27669, g8637, g8639, g8638 |
| B6GT | 5 | g8424, g12720, g12718, g12719, g24529 |
| UDPGT | 3 | g19744, g17492, g5007 |
| MYB | 2 | g23080 (MYB1), g16810 (MYB2) |

Note: A B5GT (g8639) gene is lost and a B5GT (g27669) gene is truncated in cv. Green.

Table S27. A / B compartment of each chromosomes for *A. tricolor*.

|  | methylation | | gene number | | TPM of genes | | Hi-C reads count | | A / B compartment |
| --- | --- | --- | --- | --- | --- | --- | --- | --- | --- |
|  | plus | minus | plus | minus | plus | minus | plus | minus | plus |
| Chr1 | 306,007 | 393,506 | 588 | 2,011 | 9,869.01 | 95,723.83 | 552,088 | 1,517,875 | B |
| Chr2 | 325,729 | 342,464 | 562 | 1,548 | 7,214.26 | 67,661.83 | 581,650 | 1,292,378 | B |
| Chr3 | 214,499 | 396,218 | 394 | 1,704 | 6,734.22 | 65,869.57 | 444,391 | 1,335,928 | B |
| Chr4 | 294,825 | 270,458 | 530 | 1,231 | 10,232.60 | 70,515.51 | 564,330 | 1,057,844 | B |
| Chr5 | 264,990 | 206,213 | 499 | 1,148 | 14,004.51 | 49,408.97 | 591,588 | 855,711 | B |
| Chr6 | 267,287 | 247,000 | 1,222 | 417 | 45,228.46 | 10,450.97 | 955,486 | 489,141 | A |
| Chr7 | 242,374 | 258,120 | 568 | 998 | 19,494.88 | 50,473.06 | 533,607 | 823,265 | B |
| Chr8 | 223,779 | 250,237 | 1,078 | 410 | 40,015.89 | 9,096.86 | 849,431 | 429,735 | A |
| Chr9 | 271,446 | 202,222 | 1,107 | 330 | 36,186.99 | 4,229.18 | 909,347 | 336,978 | A |
| Chr10 | 177,346 | 334,032 | 973 | 576 | 33,102.61 | 6,677.98 | 698,987 | 534,715 | A |
| Chr11 | 191,234 | 232,376 | 380 | 1,226 | 14,111.82 | 33,503.03 | 403,848 | 646,259 | B |
| Chr12 | 188,927 | 219,386 | 981 | 420 | 34,395.18 | 9,913.11 | 760,264 | 412,881 | A |
| Chr13 | 187,989 | 205,191 | 1,020 | 473 | 52,300.79 | 9,143.89 | 828,725 | 462,208 | A |
| Chr14 | 247,175 | 164,596 | 451 | 848 | 12,761.62 | 32,782.18 | 489,922 | 678,798 | B |
| Chr15 | 300,001 | 124,473 | 447 | 687 | 8,394.30 | 38,755.46 | 515,599 | 516,736 | B |
| Chr16 | 200,917 | 163,310 | 823 | 305 | 28,903.41 | 3,344.76 | 688,375 | 226,513 | A |
| Chr17 | 159,372 | 114,329 | 858 | 416 | 44,059.54 | 16,079.38 | 669,461 | 345,090 | A |

Note: A compartments are active regions of chromosomes, and usually have less methylation, more gene number, higher gene expression and less Hi-C interaction. Plus and minus mean scores of PCA analysis for each chromosome.

Table S28. Data sources of relative species.

|  | Download sites |
| --- | --- |
| *A. cruentus* | <https://bioinformatics.psb.ugent.be/gdb/amaranthus/> |
| *A. palmeri* | <https://genomevolution.org/coge/GenomeInfo.pl?gid=56750> |
| *A. tuberculatus* | https://genomevolution.org/coge/GenomeInfo.pl?gid=51756 |
| *A. hypochondriacus* | https://genomevolution.org/coge/GenomeInfo.pl?gid=34733 |
| *A. hybridus* | https://genomevolution.org/coge/GenomeInfo.pl?gid=57429 |
| *B. vulgaris* | https://www.ncbi.nlm.nih.gov/data-hub/genome/GCF_000511025.2/ |
| *C. quinoa* | https://www.ncbi.nlm.nih.gov/data-hub/genome/GCF_001683475.1/ |
| *S. oleracea* | https://www.ncbi.nlm.nih.gov/data-hub/genome/GCF_002007265.1/ |
| *H. undatus* | http://www.pitayagenomic.com/download/ |
| *S. chinensis* | https://download.cncb.ac.cn/gwh/Plants/Simmondsia_chinensis_sc1_GWHAASQ00000000/ |
| *D. caryophyllus* | http://carnation.kazusa.or.jp/ |
| *A. vesiculosa* | https://www.biozentrum.uni-wuerzburg.de/carnivorom/resources |
| *F. tataricum* | http://mbkbase.org/Pinku1/ |
| *A. thaliana* | https://www.ncbi.nlm.nih.gov/data-hub/genome/GCF_000001735.4 |

Supplementary References:

**Jumper J, Evans R, Pritzel A, Green T, Figurnov M, Ronneberger O, Tunyasuvunakool K, Bates R, Zidek A, Potapenko A, et al. 2021.** Highly accurate protein structure prediction with AlphaFold. *Nature* **596**(7873): 583-+.

**Kurtz S, Phillippy A, Delcher AL, Smoot M, Shumway M, Antonescu C, Salzberg SL. 2004.** Versatile and open software for comparing large genomes. *Genome Biology* **5**(2).

**Nattestad M, Schatz MC. 2016.** Assemblytics: a web analytics tool for the detection of variants from an assembly. *Bioinformatics* **32**(19): 3021-3023.

**Osuna-Cruz CM, Paytuvi-Gallart A, Di Donato A, Sundesha V, Andolfo G, Aiese Cigliano R, Sanseverino W, Ercolano MR. 2018.** PRGdb 3.0: a comprehensive platform for prediction and analysis of plant disease resistance genes. *Nucleic Acids Res* **46**(D1): D1197-D1201.

**Shumate A, Salzberg SL. 2021.** Liftoff: accurate mapping of gene annotations. *Bioinformatics* **37**(12): 1639-1643.
